# Practice nurse management of hypertension in primary care: a scoping review

**DOI:** 10.1017/S1463423626101455

**Published:** 2026-08-11

**Authors:** Luke Heath, Ann Smith

**Affiliations:** School of Health and Social Wellbeing, University West of England, Bristol, UK

**Keywords:** hypertension, nursing remit, primary care nursing, primary health care

## Abstract

**Aim:**

To review the current diagnosis and treatment of hypertension (HTN) and explore alternative care management options.

**Background:**

HTN is underdiagnosed and poorly managed in the UK. With an increasing emphasis on teamwork in primary care (PC), what is the evidence base supporting the partial or complete management of HTN by practice nurses (PNs)?

**Methods:**

Using the PRISMA-Scr checklist as a framework, six database searches (BNI, CINAHL, Embase, Emcare, Medline, and PubMed) were conducted using Boolean Operators between December 2023 and February 2024, employing four search structures to find literature on independent or collaborative HTN management by PNs. Qualitative and quantitative research studies, reports, and articles were all considered.

**Findings:**

HTN is a significant risk factor for mortality and morbidity. Despite clear national guidelines on the diagnosis and management, many patients affected by this condition remain undiagnosed, with the majority of those diagnosed inadequately treated. PC is struggling to manage the workload due to an ageing population and reduced general practitioner (GP) numbers. The development of new roles to support GPs in their work is underway, but current roles remain vague and without nationally agreed remits or working practices. PNs currently manage most chronic non-communicable diseases, such as diabetes, asthma, and chronic obstructive pulmonary disease; however, HTN is not typically part of their remit. There is strong quantitative and qualitative evidence supporting PNs’ involvement in HTN management as care coordinators. Such a move could improve patient outcomes and release GP time, but institutional barriers need to be addressed.

## Background

### Introduction

Hypertension (HTN), or persistently elevated blood pressure, is a significant cause of mortality and morbidity in the general population. It independently increases the risk of comorbidities such as heart failure, stroke, kidney disease, and vascular dementia (NICE - CKS, 2023). It affects around 32% of adults in England, with an estimated 29% (or 4.2 million people) remaining undiagnosed (ONS, 2023). Once identified, clear national guidelines recommend lifestyle changes, medication selection and dose titration to achieve blood pressure targets. However, substantial variation in HTN clinical management is observed in England, with diagnosed and treated patients only achieving the target range between 11.8 and 47.9% of the time (Coyle *et al*., 2019). A similar pattern has been observed in other countries (Vay-Demouy *et al*., 2023). This indicates that the majority of patients diagnosed with the condition face ongoing associated risks due to suboptimal treatment.

The screening, assessment, diagnosis, and management of HTN are mainly carried out in primary care (PC) in the UK. Although approximately 90% of all NHS contacts occur in PC (Donaldson *et al*., 2021), the increasing demand from an ageing population has coincided with a fall in the number of general practice doctors due to various reasons outlined in the ‘Long Term Plan’ (NHS, 2019:13). It was predicted that the NHS in England alone will have 7,000 fewer full-time equivalent general practitioners (GPs) in 2024 than in 2019 (Beech *et al*., 2019). Historically, a GP would manage most of a patient’s acute and chronic health problems; this is no longer possible. There are calls to expand the team to include new clinical roles (Beech *et al.*, 2019, figure 2) to improve access and capacity.

The situation is not unique to the UK, and various options are being explored globally (Freund *et al*., 2015), with a focus on team care rather than delegation of tasks from the GP to the team (task-oriented care). Team care has the potential to improve standards by providing care that is ‘tailored, frequent, and collaborative’ (Mills *et al*., 2018, 8). The roles in this multidisciplinary team are diverse (Carrier, 2023, chapter 3). They include existing staff such as practice nurses (PNs), nurse practitioners (NPs), and paramedics (Chouinard *et al*., 2017), and new roles such as physician associates (PAs) or prescribing pharmacists.

NHS England has promoted the development of these new roles in PC through funding under the Additional Roles Reimbursement Scheme (Khan, 2022). However, new positions, such as the PA, introduced in the UK in 2003, still only numbered 1,149 as of 2022 (NHS England, 2022). This is despite the additional funding they attract. They also cannot prescribe medication and must work under GP supervision. In the same year, there were over 4,900 nurses and health visitors with a job description of ‘Advanced Practitioner’ (William *et al*., 2023) covering both primary and secondary care. This contrasts with the 23,300 (16,947 FTE) PNs already in post in England (NHS Digital, 2024).

While future pharmacy graduates will be able to prescribe at the point of registration from 2026 (NHS England, 2024), currently most pharmacists working in PC do not prescribe and their role is one of medication reconciliation and review. Most paramedics and NPs are employed to manage acute on-the-day demand.

**Figure 1.**
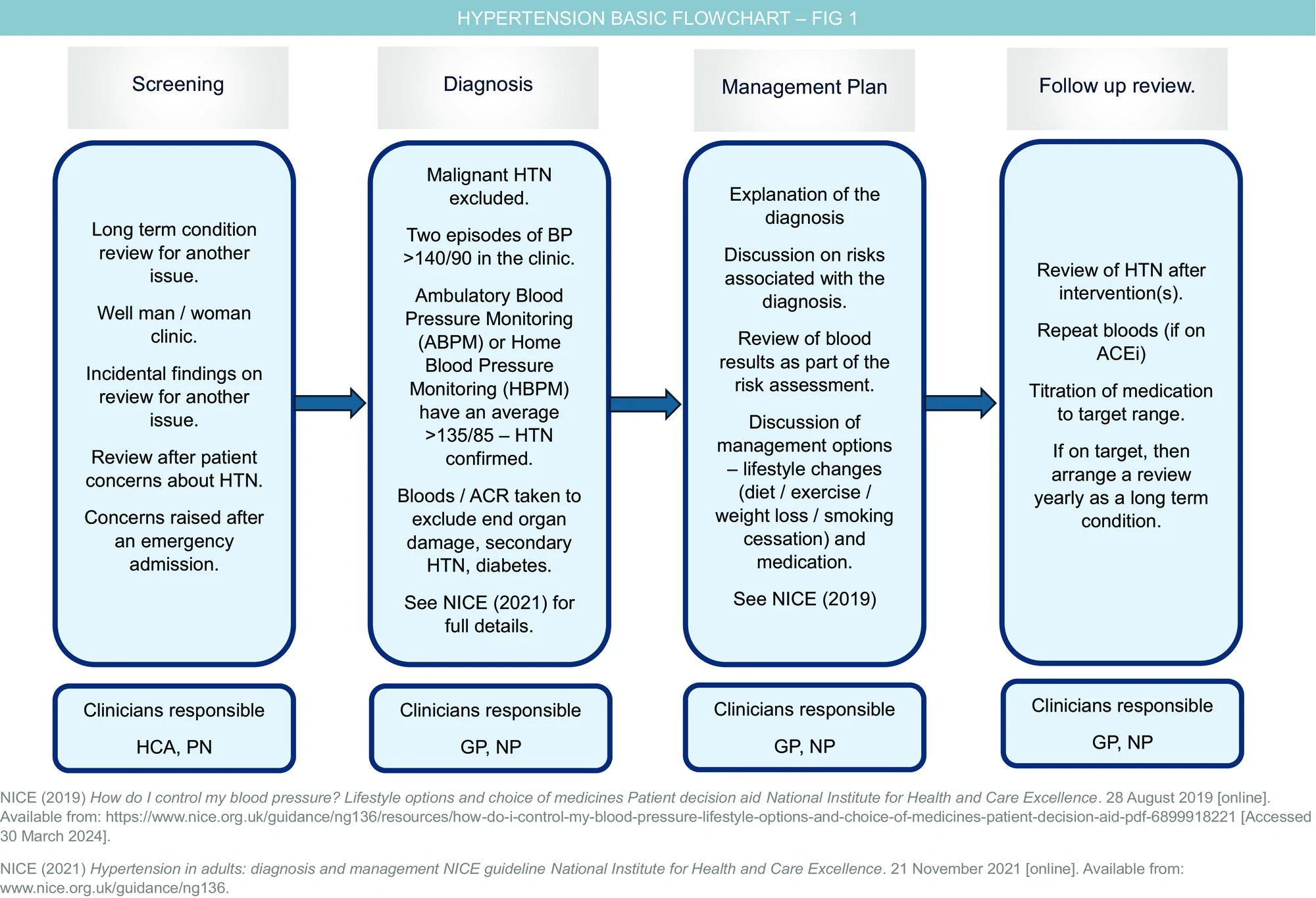
Figure 1 long description. Summary of hyptension screening and management.

The management of HTN by nurses is not routinely part of practice in PC in the UK. Where nurses are managing HTN, then it is likely that they are NP’s (Figure 1) and Health Education England lists HTN as part of their responsibilities (May *et al*., 2020). However, most nurses in PC are PNs. They have completed post-registration qualifications to manage childhood immunisations, cervical screening, and wound care, with additional courses to independently manage conditions such as diabetes, asthma, chronic obstructive pulmonary disease (COPD), and contraception.

There is substantial evidence that clinical task shifting to nurses can be achieved safely and effectively. Multiple Cochrane reviews demonstrate that nursing care is equivalent to, or better than, GP care for many conditions, with improved mortality, clinical outcomes, and patient satisfaction (Kuethe *et al*., 2013; Weeks *et al*., 2016; Laurant *et al*., 2018; Karimi-Shahanjarini *et al*., 2019). However, there is a lack of clarity on the academic levels and clinical roles of nurses, both nationally and internationally.

In the UK the services provided by PC are delivered under an independent business model where GP teams (partners) or Limited Company organizations are commissioned to provide healthcare services to a specific population. This means staff are employed under a private contract, rather than the Agenda for Change banding used in secondary care. This results in significant variation in pay, leave, clinical practice, and working conditions across the NHS in England alone (The Queen’s Nursing Institute, 2015). There are efforts to provide some clarity on the clinical remit of PN’s, which indicates that the assessment of patients and development of care plans for HTN is within the remit of a Level 5 PN (Health Education England, 2015:121).

This indicates that a significant public health issue is being poorly managed, despite clear guidelines for its care. The cause is a combination of increasing demand, labour shortages, and a lack of clear standards regarding the structure and organization of the PC team. Improving HTN care requires an agreed team structure and remit, and the PN is best placed to take on a key part or all of this role. As this represents a change to current practice, a review is necessary to evaluate the efficacy, safety, remit, and patient opinion on this change.

### Objectives

Effective teamwork can enhance clinical outcomes and patient satisfaction; however, its success depends on identifying the processes involved and assigning roles to team members. Government plans to expand the team have had a limited impact on this specific issue. A PC team will typically consist of GPs, NPs, PNs, healthcare assistants, and pharmacists. Of this team, the PN, who is already independently managing other long-term conditions (LTCs) such as diabetes and asthma, is well placed to take over HTN management. This review will look at the available evidence supporting the use of PNs in the management of HTN in PC in the UK.

## Methods

### Study protocol

This study offers a scoping review of the literature on HTN management in PC. The scoping review methodology has been selected to evaluate the evidence base for the most suitable clinicians to manage HTN, as various possible combinations exist, allowing for a comparison of the evidence supporting each. Previous Systematic Reviews have found positive outcomes from nurses, pharmacists, medical assistants, and community health workers managing HTN (Mills *et al.*, 2018). Scoping reviews provide a broad overview of the evidence, allowing mapping of the data to support the direction of future planning and research (Munn *et al*., 2018). The aim is to clarify the evidence base for PNs to manage HTN in any capacity; whether fully, partly, or in collaboration with other members of the PC team. This review does not focus on how HTN should be managed in practice, but rather on which team structure has the strongest evidence base. The Preferred Reporting Items for Systematic Reviews and Meta-Analyses Extension for Scoping Reviews (PRISMA-ScR) checklist was chosen to ensure transparency of the methods and findings (Tricco *et al*., 2018). The methods and rationale for data sampling are discussed below.

### Identifying the research question

There are clear practical reasons why HTN care could be managed by a PN. However, without prescribing rights, support from other staff members will be necessary, although they could administer medication under a patient group directive (PGD). For this reason, PNs are included in each search, collaborating with other potential team members, resulting in four search strategies combined in the final review (Appendix 1). Various terms are used for each staff group due to the absence of consensus at both national and international levels.

### Eligibility criteria and information sources

**Figure 2.**
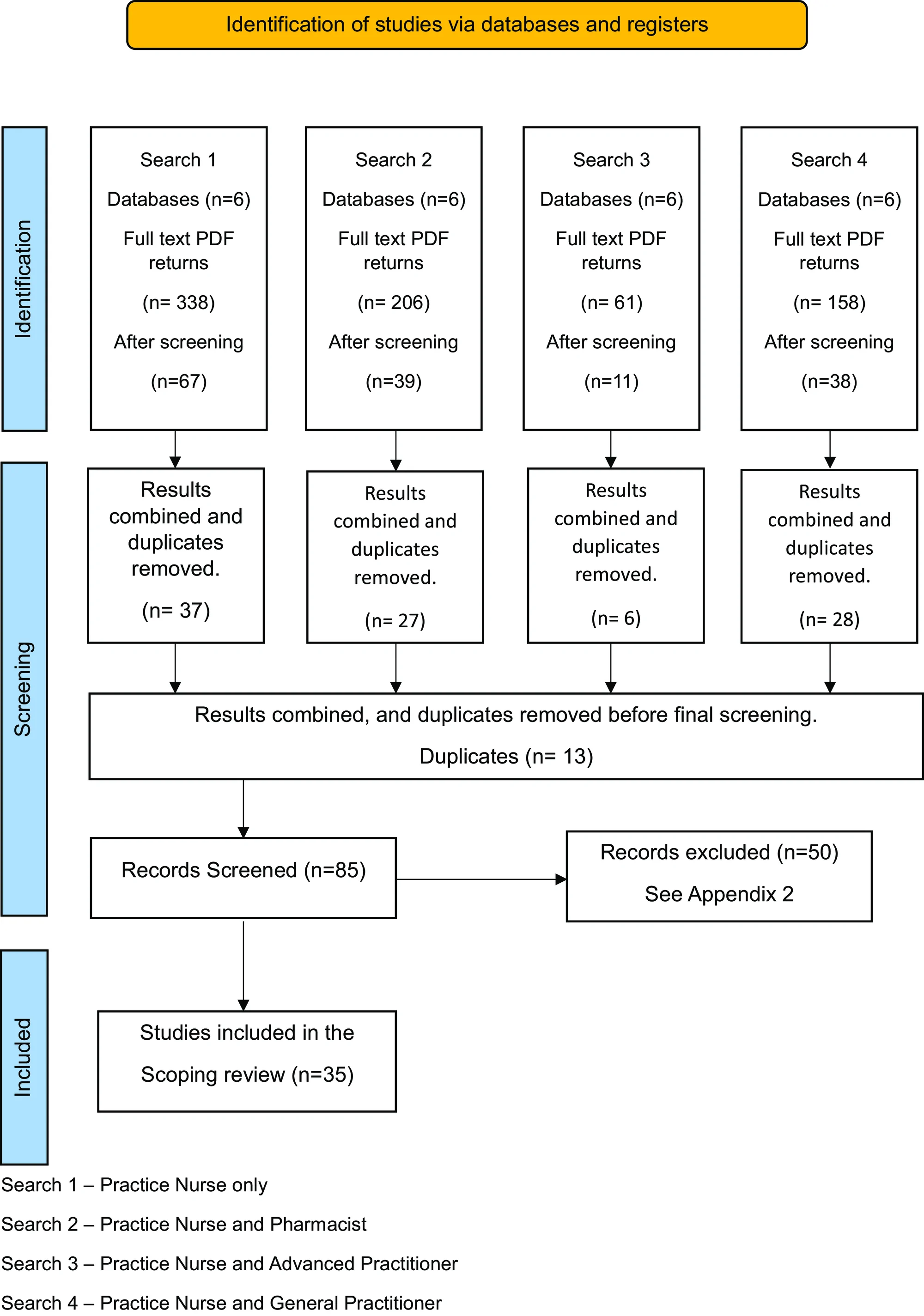
Study identification and screening flow chart.

Search limitations were in English, covered the last 10 years, were peer-reviewed, and included articles with full text availability. MESH terms were used if present for the database. A search using the Boolean phrase for independent PN HTN management (Search 1 in Appendix 1) was used as a trial since it has the fewest exclusionary criteria. The above strategy was applied to multiple databases with the following results- AMED (1), British Nursing Index (40), CINAHL (20), Embase (111), Emcare (40), HMIC (2), Medline (61), Social Policy and Practice (0), PubMed (66), and ClinicalKey (0 – no Boolean search capability). The six most productive databases – BNI, CINAHL, Embase, Emcare, Medline, and PubMed – were selected for all searches, which took place between December 2023 and February 2024 (Figure 2).

### Search and selection of sources

The four complete search strategies are listed in Appendix 1. Primary screening was conducted using the article summaries. For articles that appeared appropriate but lacked a full-text PDF with OpenAthens access, a more specific search was performed using the University of the West of England Library services and online in Google Scholar. Where a full text could not be located, the article was discarded as its relevance could not be confirmed. All articles that appeared relevant and had a full-text PDF available were further screened. The rejected articles can be found in Appendix 2, with a rationale for each exclusion, such as lack of data or role specifics.

## Results

### Data charting

The relevant studies were summarized and charted by author, year, country, study type, sample size, relevance to the question, and observations on how the findings relate to the question. These results are presented in Appendix 3, with a further summary shown in Table 1. Due to the broad scope of the studies and the diversity of methods and objectives, formal evidence mapping or grading is challenging. An additional review of the qualitative articles included has been conducted to explore staff and patient opinions on the specific question, as detailed in Appendix 4.

**Table 1.** Final articles evidence summary Table 1 long description.

Study	Country	Numbers	Type	Type of evidence	GP or team care	Practice nurse care?	Practice nurse	Pharmacist	Nurse practitioner
Maheux *et al.* (2014)	Canada	186	GP’s	Qualitative	GP’s	No			
O’Neill *et al.* (2014)	United States	126	Patients	Non-randomized retrospective comparison	Either	Partly	Case management	Medication management	
Deales *et al*. (2014)	Italy	726	Patients	Cluster-randomized control trial	Team	Partly	Case management		
Martínez-González *et al.* (2014)	Switzerland	1,344	Patients	Systematic review and meta-analysis	Team	Yes	Full care		
Allen *et al.* (2014)	United States	525	Patients	Randomized control trial	Team	No			Full care
Rumball-Smith *et al.* (2014)	Canada and New Zealand	330	Clinics	Questionnaire	Team	No	No		
Proia *et al.* (2014)	United States	52	Studies	Systematic review	Team	Partly	Case management	Case management	
Fortuna *et al.* (2015)	United States	1,300	Patients	Intervention trial	Team	Partly	Case management	Medication management	
Carrington and Stewart (2015)	Australia	530	Patients	Observational study	Either	Partly	Life coaching		
Hussain *et al*. (2015)	United States	39	GP’s + NP’s	Qualitative	Team	Partly	Case management		
Weinstein and Rucker	United States	4,021	Patients	Intervention trial	Team	Yes	Full care		
Price-Haywood *et al.* (2017)	United States	1,111	Patients	Observational Study	Team	No		Case management	
Matthys *et al.* (2017)	Belgium	13,165	Patients	Systematic review	Team	Partly	Case management		
Ciapponi *et al.* (2017)	Cochrane Library - International	77,245	Patients	Overview of systematic reviews	Team	Yes	Full care		
Lukewich *et al.* (2018)	Canada	24	Clinics	Cross-sectional survey	GP	No			
Gorina *et al*. (2018)	Spain	15,439	Patients	Systematic review	Team	Partly	Life coaching		
Derington *et al.* (2019)	United States	55,920	Patients	Cost-effectiveness analysis	Team	Yes	Case management	Case management	
Schor *et al*. (2019)	Israel	516	Clinics	Cross-sectional survey	Team	Yes	Case management		
Girvin (2019)	UK			Journal article	Team	Yes		Full care	
Lee *et al.* (2019)	United States	4,762	Patients	Intervention trial	Team	Yes	Case management		
Ödesjö *et al*. (2019)	Sweden	88,945	Patients	Retrospective cohort study	Team	Yes	Case management		
Marco-Moreno *et al.* (2020)	Spain	8	Patients	Qualitative	Team	Partly	Case management		
Perris (2020)	United States			Journal article	Team	Partly			
John *et al*. (2020)	United States – international studies used	60,617	Patients	Systematic review and meta-analysis	GP’s	No			
Lin *et al*. (2020)	United States	717	Patients	Intervention trial	GP’s	No			
Van Grondelle *et al.* (2022)	Netherlands	15	GP’s + PN’s	Qualitative	Team	Partly	Case management		
Nunes Oliveira *et al.* (2022)	Brazil	23	GP’s + NP’s	Qualitative	Team	Partly	Case management		
Stephen *et al.* (2023a)	Australia	65	Patients	Cluster-randomized control trial	Team	Yes	Case management		
Weise *et al*. (2023)	Germany	17	Patients	Qualitative	Team	Yes	Case management		
Hellgren *et al.* (2023)	Sweden	76	Clinics	Clinical management review	Team	Partly	Case management		
Stephen *et al*. (2023b)	Australia	8	GP’s + PN’s	Qualitative	Team	Yes	Case management		
Martínez-Mardones *et al*. (2023)	Chile	324	Patients	Cluster-randomized control trial	Team	No		Case management	
Yu *et al*. (2023)	Hong Kong	212,707	Patients	Prospective cohort study	Team	Yes	Case management		
Stojnić *et al*. (2023)	Slovenia	48	GP’s + PN’s	Qualitative	Team	Partly	Case management	Medication management	
Anand (2024)	United States	48	Patients	Quality improvement project	Team	Yes	Case management		

### Selection of sources

The total number of search results was 763. After excluding irrelevant material, the number of articles was reduced to 155 full-text articles for review. Removing duplicates from each search left 98 articles. Combining all search results and eliminating duplicates resulted in 85 studies. Each study was evaluated with the following question: Does this provide evidence to support changes in care, new ways of working, or modifications to the structure of clinical care? A further 50 articles were excluded after full-text review, as listed in Appendix 2, leaving 35 articles for final review and analysis. See Figure 2.

### Synthesis

#### Distribution of studies

**Figure 3.**
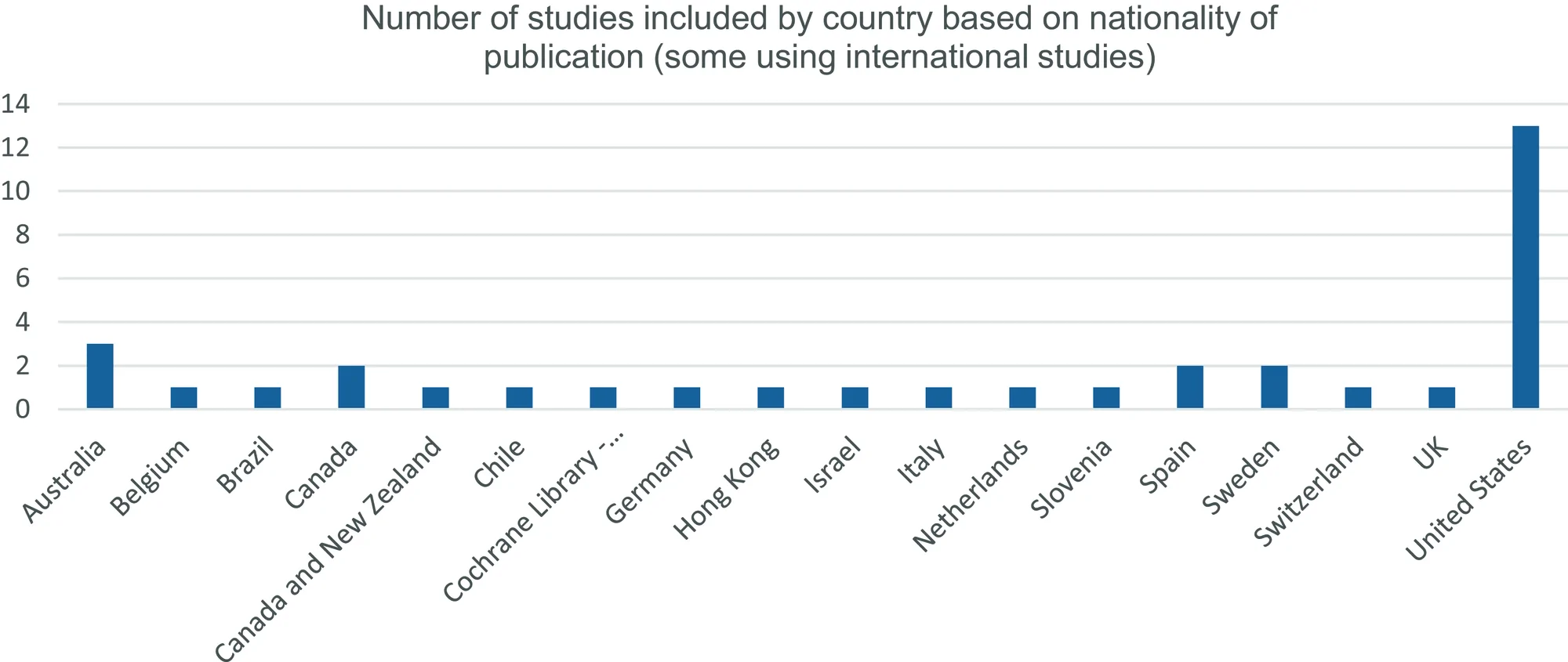
Study numbers by nationality.

Only one article was from the UK. This imposes significant limitations on the interpretation, as staff roles and scope of practice are not universal. 13 studies were published in the United States, where there is some arguable overlap between a NP in the United States and the UK (Figure 3). However, many states in the United States impose limitations on the full scope of practice compared to other states. The role of PN in the United States appears unclear, with different roles such as PAs and physician assistants also mentioned as fulfilling similar remits. Within Europe, roles such as PN are not fully defined or universal, and countries like Germany and Slovakia do not appear to have NPs (Stojnić *et al*., 2023; Weise *et al*., 2023).

#### Identification of roles

Roles are described in very different ways and are not easily interchangeable. When the practice area was PC and the staff member was called a registered nurse (as opposed to a nursing auxiliary or nursing assistant), this was interpreted as being equivalent to a PN in the UK. Any registered nurse with independent prescribing rights or who operates independently as part of their role is considered comparable to an NP in the UK. The rationale for each decision is provided in Appendix 3. When the term ‘pharmacist’ was used, it was understood to mean a non-prescribing pharmacist unless prescribing was explicitly included in their role, in which case they are regarded as equivalent to a clinical pharmacist (CP) in the UK. Many of these terms are not fully standardized or universally accepted in the UK and are used here for clarity in comparison.

## Summary of evidence

### Qualitative studies

**Figure 4.**
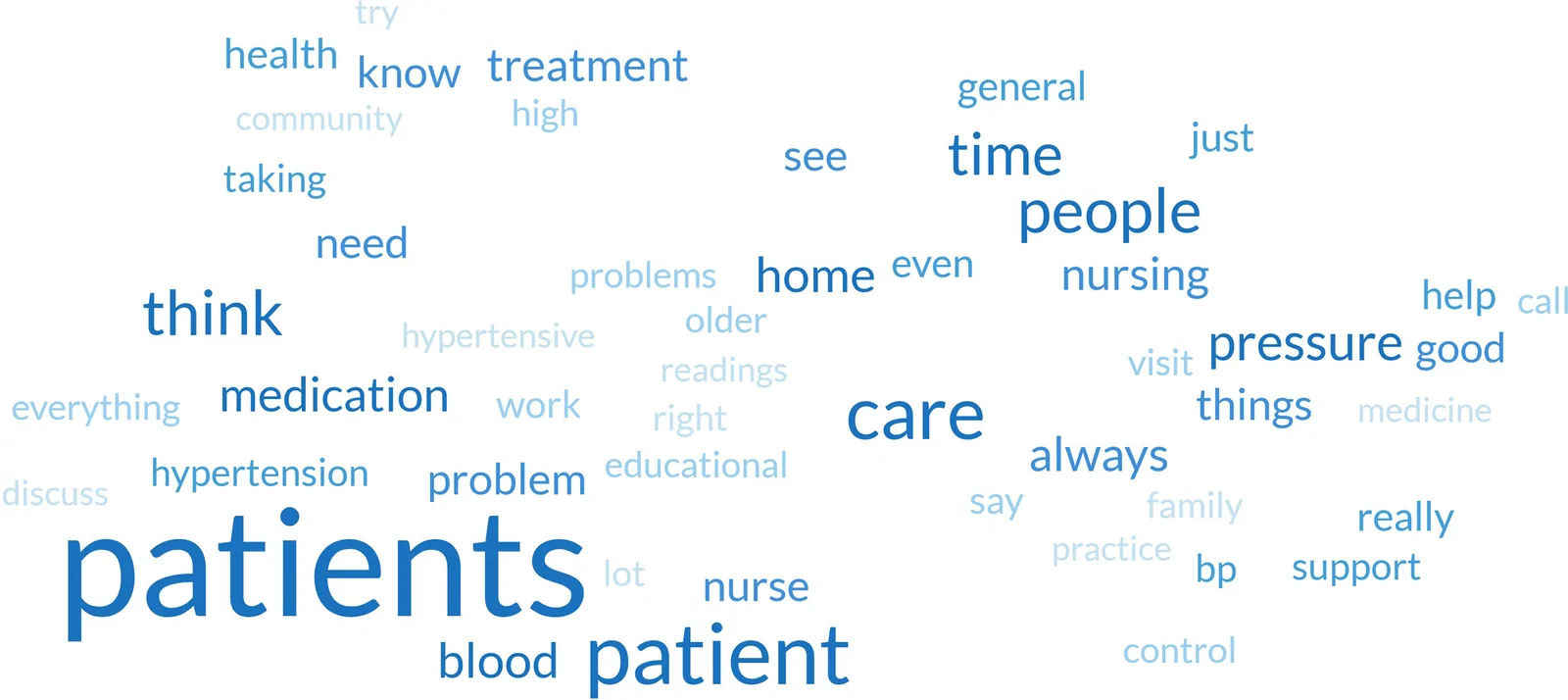
Word cloud from all quotes in qualitative reviews.

There is a broad body of evidence, as expected from a scoping review. Eight qualitative reviews involve various staff and patient groups (Appendix 4). They include online surveys, structured interviews, and focus groups. These identify common themes, and the patient’s opinion is incorporated. Figure 4 shows a word cloud of all quotes from the qualitative reviews, whether from the article or the tables. It indicates that the patient remains central to most discussions.

Staff agree that the current system is ineffective and that there is a ‘need for change’ (Stephen *et al*., 2023b). The main identified change is that patients require more time for support and education on HTN. This approach provides patients with a sense of security, helps them understand the importance of medication compliance, and encourages them to take a symptom-free condition seriously.

Patients were perceived as being dependent on the healthcare system and not taking responsibility for self-care (Hussain *et al*., 2015; Stojnić *et al.*, 2023) There were concerns about the risks to patients caused by the current poor HTN management (Hussain *et al*., 2015), and that this might be leading to unnecessary secondary care referrals (Van Grondelle *et al*., 2022).

Team-based care was believed to be more effective than GP care alone (Hussain *et al*., 2015; Marco-Moreno *et al*., 2020; Van Grondelle *et al*., 2022; Stojnić *et al.,* 2023; Weise *et al.*, 2023). This team included various roles, from PNs, pharmacists, and life coaches to social services and the patient themselves. While team care is seen as a solution to creating more time for patients, and patients see PNs as trusted and more accessible than GPs (Weise *et al*., 2023), there is no clear consensus on who should take on specific roles. Nunes Oliveira *et al*. (2022) preferred a collaborative team model with shared horizontal decision-making, while Stojnić *et al.* (2023) saw the GP as the care coordinator in a clear hierarchy, with an expanded team to support them in this role.

GPs were generally receptive to allowing nurses to titrate medication for HTN, although there were reservations about granting them greater independence. Two studies reported resistance by GPs to expanding the nursing scope of practice (Maheux *et al*., 2014; Stojnić *et al*., 2023). However, patients expressed strong support for the expansion of PN remit beyond typical boundaries (Weise *et al.*, 2023). Patients raised concerns that team-based care could reduce GP numbers, create barriers to accessing a GP or that PN’s would expect their salaries to rise (Weise *et al*., 2023). Marco-Moreno *et al*. (2020) identifies that GP support for significant change (in this case, patient self-titration of medication) was dependent on participating in the study that demonstrated improved outcomes, suggesting resistance could be reduced with GP education.

### Quantitative studies

Among the remaining quantitative studies, there was strong evidence supporting team care as being better than GP care in outcomes, which was expected. Table 2 provides a summary of each study type, the number of participants involved, and the expression of support for six identified potential outcomes.

**Table 2.** Summary of study type and number of participants Table 2 long description.

Types of study	Team care	GP care	PN full care	PN case management	PN life coaching	Pharmacist med management	Pharmacist case management
Clinical management review	76	0	0	76	0	0	0
Cluster-randomized control trial	1,115	0	0	791	0	0	324
Cost-effectiveness analysis	55,920	0	0	55,920	0	0	55,920
Cross-sectional survey	516	0	0	516	0	0	0
Intervention trial	10,083	717	4,021	6,062	0	1,300	0
Journal article	0	0	0	0	0	0	0
Non-randomized retrospective comparison	0	0	0	126	0	126	0
Observational study	1,111	0	0	0	530	0	1,111
Overview of systematic reviews	77,245	0	77,245	0	0	0	0
Prospective cohort study	212,707	0	0	212,707	0	0	0
Qualitative	141	186	0	158	0	48	0
Quality improvement project	48	0	0	48	0	0	0
Questionnaire	330	0	0	0	0	0	0
Randomized control trial	525	0	0	0	0	0	0
Retrospective cohort study	88,945	0	0	88,945	0	0	0
Systematic review	28,656	0	0	13,217	15,439	0	52
Systematic review and meta-analysis	1,344	60617	1,344	0	0	0	0
Totals	478,762	61,520	82,610	378,566	15,969	1,474	57,407

The figures used reflect the volumes involved in each study or trial. Some represent the patients involved in a clinical trial, while others denote the staff engaged in a survey. They aim to provide context on the scale and significance of the study referenced. Where an article examines multiple clinical questions, only the figures from those studies related to the question have been included, as in Martínez-González *et al*. (2014). When a systematic review includes studies, but no figures are provided, and the referenced studies cannot be located, the total number of studies has been used, as in Proia *et al*. (2014). This enables the conclusion of the study to be represented, although its impact is diminished compared to other studies where subject numbers are provided.

### Team care

**Figure 5.**
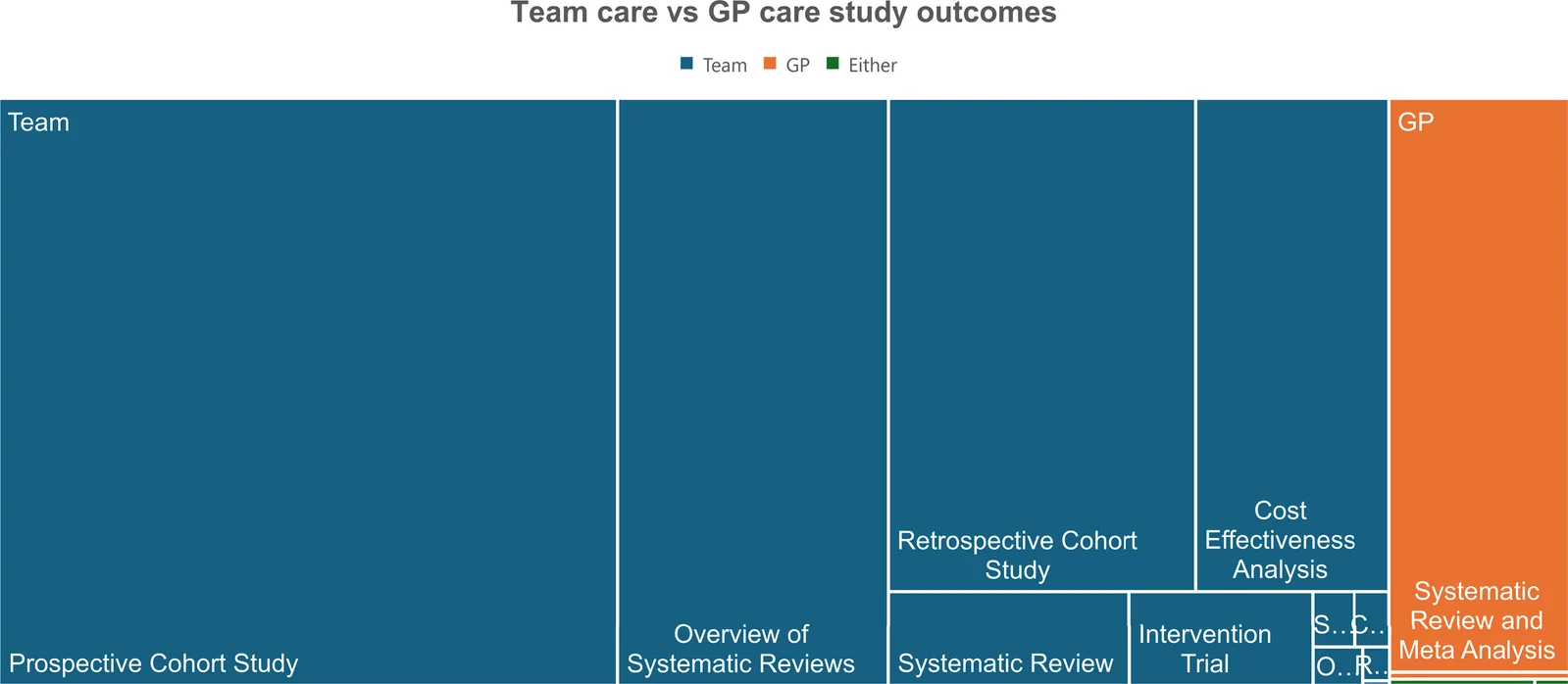
Team working versus GP care study outcomes Treemap.

Team care was generally regarded as superior to GP care alone, although two studies found it less effective. The Matthys *et al*. (2017) review was a comprehensive systematic review that included 285 primary studies containing 173 RCTs, which examined PN and GP collaboration for multiple LTCs. Many outcomes were mixed; some were slightly worse, with only HTN exclusively found to be improved in three reviews. John *et al*. (2020) contradicts this with a systematic review of 85 trials involving 60,617 patients, comparing team care to GP care. The review found that team care was significantly better for LTCs, except for HTN, where the improvements were not statistically significant. They caution the findings due to the high heterogeneity of the outcomes (17). Overall, the case for team working in the management of HTN is strong, with large numbers in a variety of trials that focused on patient outcomes (Figure 5)

The most common team composition was the PN and the GP (11 trials), of which two (John *et al*., 2020; Lin *et al*., 2020) found little or no benefit. The remainder were positive (Deales *et al*., 2014; Proia *et al*., 2014; Carrington and Stewart, 2015; Fortuna *et al*., 2015; Matthys *et al*., 2017; Ödesjö *et al.*, 2019; Schor *et al*., 2019; Yu *et al*., 2023; Stephen *et al*., 2023a). Two studies included a GP and a pharmacist; Price-Haywood *et al*. (2017) was not supportive of CP management, while Martínez-Mardones *et al.* (2023) was supportive of pharmacist management.

### Practice nurse care

Regarding the specific question of managing HTN by PNs, out of the 35 reviews, 27 either directly or partly addressed the question; the remaining were supportive of increasing PN clinical autonomy. Fourteen studies superficially endorsed PNs to do this in a collaborative role (Deales *et al*., 2014; Martínez-González *et al.*, 2014; O’Neill *et al.*, 2014; Carrington and Stewart, 2015; Matthys *et al.*, 2017; Gorina *et al*., 2018; Ödesjö *et al.*, 2019; Schor *et al*., 2019; Lin *et al.*, 2020; Hellgren *et al*., 2023; Yu *et al.*, 2023; Stephen *et al*., 2023a, 2023b; Anand, 2024). This is either with GPs, NPs, or CPs forming the team. In most cases, the PN acted as the ‘case manager’, providing education, support, collecting data, arranging follow-up, and conducting some investigations, while the other team members made the diagnosis and prescribing decisions.

Some evidence supporting PN case management is robust, with Ödesjö *et al.* (2019) identifying that nurse-led care had a 10% higher incidence of achieving HTN control (*n* = 88,945) compared to usual GP care. This is supported by Deales *et al.* (2014), who demonstrated a 16% improvement in achieving target BP (*n* = 726) compared to GP care. A five-year review of over 200,000 patients with nurse-managed HTN showed an 8% reduction in cardiovascular disease, 10% reduction in all-cause mortality and decreased secondary care usage (Yu *et al*., 2023).

Matthys *et al.* (2017) support this with an extensive systematic review of RCTs (*n* = 173), which was not clearly supportive of collaborative care for multiple other LTCs but was for HTN. In their RCT Martínez-González *et al*. (2014) supported PN care based on outcomes better or equivalent to those of a GP, for multiple LTCs apart from HTN, but this was due to the HTN trials using NPs instead.

**Figure 6.**
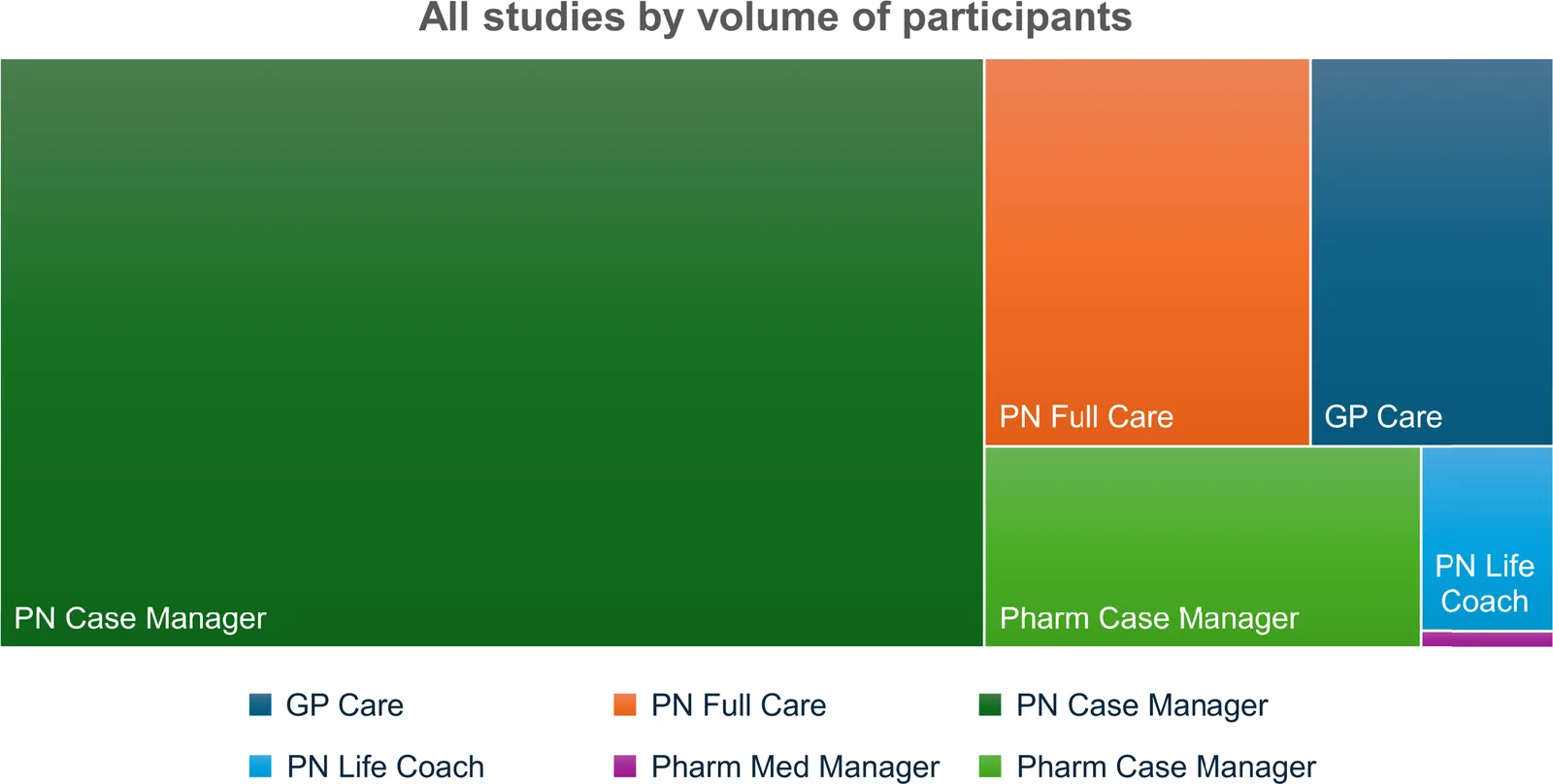
Supported outcomes shown by total number of participants in the study.

Lin *et al.* (2020) was supportive of PN collaboration in their intervention trial (*n* = 717), but outcomes were not improved over usual care, and Hellgren *et al.* (2023) suggests that PNs mainly manage HTN in Sweden, but some elements of care appear inadequate. A further three studies, which did not directly address the question, also supported PN management of clinical conditions, with some oversight (Gorina *et al.*, 2018; Schor *et al.,* 2019; Stephen *et al*., 2023a). These studies examined nurses as educators for chronic disease and show significant improvements in patient outcomes, including HTN, from this role. Figure 6 shows a summary of the study’s subjects and the supported management option.

Two additional studies supported a PN and pharmacist team assisting the GP in the management of HTN. Fortuna *et al*. (2015) was a large intervention trial (*n* = 13,404) where 67.4% of all patients with HTN achieved their target goal, and high-risk patients increased from under 40% to around 60%. Proia *et al.* (2014) was a systematic review of 52 studies that examined having a PN, a pharmacist, or both as support for GP care, finding substantial improvements in HTN management compared to GP care alone.

Four studies support complete independent HTN management by PN’s. The small qualitative review by Weise *et al*. (2023), mentioned above, along with an article by Perris (2020), which shows how PNs working under standardized procedures managed multiple clinical conditions, including independently providing medication. In a small trial involving 4,021 patients, Weinstein and Rucker found that a PN-led ‘Treat to Target’ programme improved HTN control from 40% to 62% with lifestyle support and medication titration all part of the PN remit.

Cost implications were examined in some studies, suggesting nursing care is more expensive due to increased investigations and medication being issued (Allen *et al*., 2014) making teamworking more effective but expensive (Schor *et al*., 2019). This could indicate a situation where an increased number of reviews by a cheaper member of staff results in no overall savings. Proia *et al*. (2014) concludes that team-based care is cost-effective, improves outcomes, increases patient satisfaction and reduces per capita costs.

Lee *et al*. (2019) found the improvements in HTN were achieved without additional funding. Derington *et al*. (2019) looked at 100 trials involving over 55,000 patients to assess the cost-effectiveness of team-based care and concluded that non-physician care and medication changes were the most effective arrangement, both in clinical outcomes and cost. Yu *et al.* (2023) in their 5-year follow-up demonstrated a reduction in secondary care usage and all-cause mortality from intensive nurse management of HTN, with a reduction in adverse outcomes of 14.5% (Table 3). These downstream costs clearly need to be considered as part of the overall picture, as HTN management is preventative care.

**Table 3.** Summary of study type participants supportive of GP or team care composition Table 3 long description.

Summary of evidence	Team care	GP care	PN full care	PN case management	PN life coaching	Pharmacist medication mangement	Pharmacist case management
Patient studies	477,655	61,334	82,610	377,789	15,969	1,426	57,355
GP studies (GP’s)	0	186	0	0	0	0	0
GP’s + NP’s studies	62	0	0	62	0	0	0
GP’s + PN’s studies	71	0	0	71	0	48	0
Clinics/GP surgeries	922	0	0	592	0	0	0
Patient studies (no numbers)	52	0	0	52	0	0	52
Total	478,762	61,520	82,610	378,566	15,969	1,474	57,407

## Discussion

There are several limitations with the data. Primarily, UK studies are absent (there is just one article). When roles and responsibilities are not clearly transferable, there is a loss of context about the intervention, its relevance to current practice, and the anticipated benefits. There is also a need to interpret the results of some studies, which leads to the inclusion (or exclusion) of certain articles here that others might have chosen differently. Additionally, this period covers the COVID pandemic, and there could be a case for including older research to broaden the scope, considering the likely two-year hiatus in conducting patient studies.

All papers agree that HTN is a significant and growing concern for community health care and that the medical team is poorly managing it in most countries. Lee *et al*. (2019) calculates that taking a cohort of 628 people from 67% in the target blood pressure to over 80% would prevent nine strokes and six heart attacks, of which five would be fatal. The evidence that many PC practices are achieving less than 50% of their patients in target is a significant concern. It presents a future personal, medical, and economic burden on an already struggling health system in the UK.

There is minimal conflicting data on the proposal that task shifting specific medical responsibilities to nurses can lead to significant improvements in clinical parameters, patient satisfaction, and cost-effectiveness. The evidence suggests that, at worst, team care will match the quality of current outcomes. Matthys *et al*. (2017) agree that most of the studies they reviewed showed nursing care had added value, even if there were no clinical improvements, but cautioned that these results were most likely because nurses conducted most of them.

In the UK, the focus is on the reduced number of GPs. Therefore, the emphasis should be on removing care activities from the GP workload that others can safely manage. HTN, with its clear protocols for diagnosis and treatment, is a suitable option. However, most trials involved the nurse as the case manager under the clinical supervision of the GP. There is little evidence to support other specific team members taking over as clinical supervisors and prescribers, although they clearly could, as they do currently with other LTCs.

The evidence supporting PNs as a case manager for HTN is strong, patient-endorsed and shows an increase in the number of individuals reaching their target range. PNs working alongside a GP were more effective than a pharmacist in the same role. Independent management of HTN by PNs is not well supported, although many trials appeared to use care and medication protocols that allowed for significant clinical discretion. No trials report using PGDs for medication or similar approaches.

GP resistance was identified, which is relevant to the UK, where the GP can act as both employer and manager for partner practices. However, concerns from doctors should not prevent the implementation of best practice. The evidence shows that even non-clinical management improves outcomes, with patient self-titration of medication being more effective than GP care in small studies. It is unreasonable to argue that a PN with additional training could be inferior to a GP when the evidence does not support this.

This raises the question of whether PNs would want to assume this role. While the qualitative reviews suggest nurses are aware that the care provided is not adequate, there was nothing to indicate they were eager to take it on. Caution is required to prevent prescriptively assigning them the role when it is not the usual practice. It would, however, seem logical for them to manage it alongside other LTCs, providing better integration with other conditions associated with cardiovascular risk, such as diabetes and lipid management. A clear definition of their overall remit would be necessary to reduce concerns about further scope expansion. This introduces the risk of adverse outcomes for this staff group, such as increased stress and burnout, which need to be monitored.

This lack of UK research is intriguing, especially considering the clear efforts to create new roles and expand existing ones to meet PC pressures. It is reasonable to expect that trials would be used to reinforce and support new working practices, thereby enhancing adoption and driving change. The absence of recent PC research within the UK, even regarding established staff roles, is notable. It is particularly surprising that a large group of highly skilled nurses, most of whom are already independently managing multiple aspects of patient care, does not attract more study.

The quantitative data is good, and Yu *et al*. (2023) provides strong evidence for the potential systemic benefits of any changes. Suboptimal HTN control predisposes individuals to poor long-term health outcomes, which in the NHS is borne by the system. The main obstacle, however, is likely to be the structure of PC in the UK itself. Individual practices are run as independent businesses, each with its own staff contracts and agreed scope of practice. Rapid change will require top-down pressure rather than allowing each practice to independently evaluate the merits and personal costs. Improvements in patient outcomes and reductions in downstream expenses from fewer cardiovascular events may not be considered in local decision-making.

Changes in the scope of practice are likely to provoke discussions about remuneration and potentially lead to a push for more staff to manage the increased workload. Any progress will require a national framework defining the role of a PN, which could result in renegotiation of contracts and early staff retirement if they are unwilling to adapt.

Nevertheless, change is clearly necessary to reduce preventable harm to patients. Clarifying roles and remuneration at a national level would identify surgeries that are underutilizing or underpaying their staff. It would improve workforce and education planning and, if incorporated into contracts, would promote the diagnosis and management of HTN to optimize funding streams, as staff requirements for a population could be calculated. There is also a strong argument for providing some standardization to the PC model to enable research, which would then be comparative and transferable.

## Conclusion

HTN is a significant risk factor for mortality and morbidity. There are clear national guidelines on its diagnosis and management, but many people affected by this condition remain undiagnosed, and most of those diagnosed are not receiving optimal treatment. Management involves supporting lifestyle changes and considering medication, while patient involvement is essential for a condition that is often symptom-free.

PC is struggling to manage the workload due to an ageing population and patients with multiple comorbidities. New roles are being developed to support GPs in their work, but current roles remain vague and lack nationally agreed remits or working practices. Most non-communicable diseases, such as diabetes, asthma, and COPD, are now managed by PNs; however, HTN is not. There is strong quantitative and qualitative support for PNS to be involved in HTN management as care coordinators. Such a move would improve patient outcomes and free up GP time.

However, significant obstacles exist when changing staff working practices, especially without national guidelines. More research is needed in PC across the UK, along with a national agreement on care roles and responsibilities to support change and improve patient care.

## Supporting information

10.1017/S1463423626101455.sm001 Heath and Smith supplementary material 1 Heath and Smith supplementary material

10.1017/S1463423626101455.sm002 Heath and Smith supplementary material 2 Heath and Smith supplementary material

10.1017/S1463423626101455.sm003 Heath and Smith supplementary material 3 Heath and Smith supplementary material

10.1017/S1463423626101455.sm004 Heath and Smith supplementary material 4 Heath and Smith supplementary material

## Supplementary material

The supplementary material for this article can be found at https://doi.org/10.1017/S1463423626101455.

## Figure 1. Long description

A flowchart illustrating the stages of hypertension screening, diagnosis, management plan, and follow-up review. Panel A: Screening. Long term condition review for another issue. Well man/woman clinic. Incidental findings on review for another issue. Review after patient concerns about HTN. Concerns raised after an emergency admission. Clinicians responsible: HCA, PN. Panel B: Diagnosis. Malignant HTN excluded. Two episodes of BP greater than 140/90 in the clinic. Ambulatory Blood Pressure Monitoring (ABPM) or Home Blood Pressure Monitoring (HBPM) have an average greater than 135/85. HTN confirmed. Bloods/ACR taken to exclude end organ damage, secondary HTN, diabetes. See NICE (2021) for full details. Clinicians responsible: GP, NP. Panel C: Management Plan. Explanation of the diagnosis. Discussion on risks associated with the diagnosis. Review of blood results as part of the risk assessment. Discussion of management options: lifestyle changes (diet/exercise/weight loss/smoking cessation) and medication. See NICE (2019). Clinicians responsible: GP, NP. Panel D: Follow up review. Review of HTN after intervention(s). Repeat bloods (if on ACEi). Titration of medication to target range. If on target, then arrange a review yearly as a long term condition. Clinicians responsible: GP, NP.

Navigate back to Figure 1..

## Table 1. Long description

A table summarizing studies by country, numbers, type, evidence type, and practice roles. The table has 35 rows and 9 columns. The columns are labeled Study, Country, Numbers, Type, Type of evidence, GP or team care, Practice nurse care, Pharmacist, and Nurse practitioner. Each row provides details for a specific study. For example, the first row reads: Study: Maheux et al. (2007), Country: Canada, Numbers: 186, Type: GPs, Type of evidence: Qualitative, GP or team care: GPs, Practice nurse care: No, Pharmacist: No, Nurse practitioner: No. The table captures various studies conducted in different countries, with different sample sizes and types, and highlights the roles of GPs, practice nurses, pharmacists, and nurse practitioners in each study.

Navigate back to Table 1..

## Table 2. Long description

A table summarizing study types, participant numbers, and support for various outcomes in healthcare studies. The table has 21 rows and 8 columns. The columns are labeled Types of study, Team care, GP care, PN full care, PN case management, PN life coaching, Pharmacist med management, and Pharmacist case management. The rows list different types of studies and the number of participants involved in each type of study for the different care categories. Row 1: Clinical management review, 76, 0, 0, 76, 0, 0, 0. Row 2: Cluster-randomized control trial, 1,115, 0, 0, 791, 0, 0, 324. Row 3: Cost-effectiveness analysis, 55,920, 0, 0, 55,920, 0, 0, 55,920. Row 4: Cross-sectional survey, 516, 0, 0, 516, 0, 0, 0. Row 5: Intervention trial, 10,083, 717, 4,021, 6,062, 0, 1,300, 0. Row 6: Journal article, 0, 0, 0, 0, 0, 0, 0. Row 7: Non-randomized retrospective comparison, 0, 0, 0, 126, 0, 126, 0. Row 8: Observational study, 1,111, 0, 0, 0, 530, 0, 1,111. Row 9: Overview of systematic reviews, 77,245, 0, 77,245, 0, 0, 0. Row 10: Prospective cohort study, 212,707, 0, 0, 212,707, 0, 0, 0. Row 11: Qualitative, 141, 186, 0, 158, 0, 48, 0. Row 12: Quality improvement project, 48, 0, 0, 48, 0, 0, 0. Row 13: Questionnaire, 330, 0, 0, 0, 0, 0, 0. Row 14: Randomized control trial, 525, 0, 0, 0, 0, 0, 0. Row 15: Retrospective cohort study, 88,945, 0, 0, 88,945, 0, 0, 0. Row 16: Systematic review, 28,656, 0, 0, 13,217, 15,439, 0, 52. Row 17: Systematic review and meta-analysis, 1,344, 60,617, 1,344, 0, 0, 0. Row 18: Totals, 478,762, 61,520, 82,610, 378,566, 15,969, 1,474, 57,407.

Navigate back to Table 2..

## Table 3. Long description

A table summarizing study types and participants supporting general practitioner or team care composition. The table has 8 rows and 8 columns. The columns are labeled as Summary of evidence, Team care, GP care, PN full care, PN case management, PN life coaching, Pharmacist medication managemnet, and Pharmacist case management. The rows are labeled as Patient studies, GP studies (GP’s), GP’s + NP’s studies, GP’s + PN’s studies, Clinics/GP surgeries, Patient studies (no numbers), and Total. Row 1: Patient studies, 477655, 61334, 82610, 377789, 15969, 1426, 57355. Row 2: GP studies (GP’s), 0, 186, 0, 0, 0, 0, 0. Row 3: GP’s + NP’s studies, 62, 0, 0, 62, 0, 0, 0. Row 4: GP’s + PN’s studies, 71, 0, 0, 71, 0, 48, 0. Row 5: Clinics/GP surgeries, 922, 0, 0, 592, 0, 0, 0. Row 6: Patient studies (no numbers), 52, 0, 0, 52, 0, 0, 52. Row 7: Total, 478762, 61520, 82610, 378566, 15969, 1474, 57407.

Navigate back to Table 3..
