# Supplementary material for: Practice nurse management of hypertension in primary care: a scoping review
Source: Prim Health Care Res Dev. 2026 Aug 11;27:e89. doi: 10.1017/S1463423626101455 (PMC13740167; doi:10.1017/S1463423626101455)
Supplement: Supplementary file 1 [file S1463423626101455sup001.docx]

### **Appendix 1**

**Boolean search operators used in Evidence Flow Chart (Fig 2)**

Search 1

The Boolean search strategy for independent PN management is listed below.

'general practice' OR 'primary care' OR 'family practice' OR ‘primary health care’

AND

hypertension OR ‘high blood pressure’ OR ‘primary hypertension’ OR ‘HTN’ OR ‘elevated blood pressure’

AND

independent OR collaborative OR interprofessional OR team*

AND

‘general practice nurs*’ OR 'practice nurs*' OR 'primary care nurs*' OR nurs*

NOT

'advanced practitioner' OR 'nurse practitioner' OR ‘advanced practice nurse’ OR ‘family nurse practitioner’ OR 'clinical practitioner' OR ‘general practitioner’ OR ‘GP’ OR doctor OR ‘medical practitioner’ OR ‘family doctor’ OR ‘family physician’ OR ‘primary care doctor’ OR ‘clinical pharmacist’ OR ‘community pharmacist’ OR pharmacis*

Search 2

The Boolean search strategy for PN management with Pharmacist support is listed below.

'general practice' OR 'primary care' OR 'family practice' OR ‘primary health care’

AND

hypertension OR ‘high blood pressure’ OR ‘primary hypertension’ OR ‘HTN’ OR ‘elevated blood pressure’

AND

independent OR collaborative OR interprofessional OR team*

AND

‘general practice nurs*’ OR 'practice nurs*' OR 'primary care nurs*' OR nurs*

AND

‘clinical pharmacist’ OR ‘community pharmacist’ OR pharmac* OR ‘prescribing pharmacist’

NOT

'advanced practitioner' OR 'nurse practitioner' OR ‘advanced practice nurse’ OR ‘family nurse practitioner’ OR 'clinical practitioner' OR ‘general practitioner’ OR ‘GP’ OR doctor OR ‘medical practitioner’ OR ‘family doctor’ OR ‘family physician’ OR ‘primary care doctor’

Search 3

The Boolean search strategy for PN management with Nurse Practitioner support is listed below.

'general practice' OR 'primary care' OR 'family practice' OR ‘primary health care’

AND

hypertension OR ‘high blood pressure’ OR ‘primary hypertension’ OR ‘HTN’ OR ‘elevated blood pressure’

AND

independent OR collaborative OR interprofessional OR team*

AND

‘general practice nurs*’ OR 'practice nurs*' OR 'primary care nurs*' OR nurs*

AND

'advanced practitioner' OR 'nurse practitioner' OR ‘advanced practice nurse’ OR ‘family nurse practitioner’ OR 'clinical practitioner'

NOT

‘general practitioner’ OR ‘GP’ OR doctor OR ‘medical practitioner’ OR ‘family doctor’ OR ‘family physician’ OR ‘primary care doctor’ OR ‘clinical pharmacist’ OR ‘community pharmacist’ OR pharmac* OR ‘prescribing pharmacist’

Search 4

The Boolean search strategy for PN management and General Practitioner support is listed below.

'general practice' OR 'primary care' OR 'family practice' OR ‘primary health care’

AND

hypertension OR ‘high blood pressure’ OR ‘primary hypertension’ OR ‘HTN’ OR ‘elevated blood pressure’

AND

independent OR collaborative OR interprofessional OR team*

AND

‘general practice nurs*’ OR 'practice nurs*' OR 'primary care nurs*' OR nurs*

AND

‘general practitioner’ OR ‘GP’ OR doctor OR ‘medical practitioner’ OR ‘family doctor’ OR ‘family physician’ OR ‘primary care doctor’

NOT

'advanced practitioner' OR 'nurse practitioner' OR ‘advanced practice nurse’ OR ‘family nurse practitioner’ OR 'clinical practitioner' OR ‘clinical pharmacist’ OR ‘community pharmacist’ OR pharmacis*
