# Supplementary material for: Practice nurse management of hypertension in primary care: a scoping review
Source: Prim Health Care Res Dev. 2026 Aug 11;27:e89. doi: 10.1017/S1463423626101455 (PMC13740167; doi:10.1017/S1463423626101455)
Supplement: Supplementary file 2 [file S1463423626101455sup002.docx]

### **Appendix 2**

**Scoping Review Outcomes – First Screening Exclusions**

| **Study** | **Reason for exclusion** |
| --- | --- |
| (Lalonde *et al.* 2014) | Development of a management framework for HTN treatment involving multiple members of the primary and secondary care team (Table 1), but the protocol (Fig 2) remains GP-led. Not aiming to change practice. Not relevant to the question. |
| (Piper *et al.* 2014) | An examination of the methods used to diagnose HTN (Clinic / ambulatory / home) and correlation with cardiovascular risk outcomes (MI / stroke). Not relevant to the question. |
| (Mino-Leõn *et al.* 2015) | Looking at improved outcomes when pharmacists counsel those being treated for HTN as part of the primary care team. This is an adjunct to the treatment process, but it does not address who makes the diagnosis or initiates treatment, etc. It is not helpful with the question. |
| (Skar *et al.* 2015) | A review protocol for a planned review of non-registered practitioner support in managing HTN. Not relevant to the question and contains no data. |
| (Hussain *et al.* 2015) | A qualitative review of care management functions for primary care providers (GPS) when treating HTN aims to enhance effectiveness. It is suggested that care managers could be helpful, though these are not specified as RNS. A ‘PARTNER’ mnemonic is proposed – this is designed to support more effective GP-led care of HTN; other roles or resources are not included explored. |
| (Yu *et al.* 2015) | A study protocol, no data. Examining the effectiveness of an HTN management protocol, not aspects of team care or role responsibilities. |
| (Alexander 2016) | An overview of Teams Based Learning and its application in Primary Care Teams (GP / NP / PA) – Relates to questions about training the team to perform tasks, but not specifically who can or should carry out the work. |
| (Barreto *et al.* 2016) | Identifying patients most at risk of poor HTN management. Supports the additional engagement required to manage the condition but does not address the question. |
| (Dobbins *et al.* 2016) | Looking at primary care teams, how they are organised and the benefits of team working – does not clarify the roles / responsibilities for members of the team, the focus is more on MH support and how this improves the service. |
| (Eads and Maruzzella 2016) | Looking at nursing support for alcohol withdrawal. Not relevant to the question. |
| (Jackson *et al.* 2016) | Study design. No data available. Evaluating titration of hypertension treatment (pharmacological and intervention). Not pertinent to the question. |
| (Tomiak *et al.* 2016) | Assessment of risk factors for cardiovascular disease, and effects of prophylactic education and support on systolic blood pressure. Supportive of this for reducing BP, did not help BMI / lipids. Relevant to the question in the extra time that other professions can give, but does not specifically answer the question. |
| (O’Donnell *et al.* 2016) | Qualitative research into the Patient Centred Medical Home looking at stakeholder perspectives on team-based care – themes of expansion of roles are looked at but not defined in a way that would allow an idea about patient preferences for who manages their care. Does not address the question. |
| (Santschi *et al.* 2017) | Study protocol for a randomised control trial. Team-based care intervention versus usual care. No data available. A GP will maintain control over medication initiation and changes. Not relevant to the question. |
| (Hong *et al.* 2017) | Review of care management aimed at improving outcomes, including HTN, particularly focused on the BAME community – this can involve any non-physician staff, supporting the proposal but not addressing the question as there are no formal team structures. identified. Unusual outcome review looking at cost per quality-of-life improvement. |
| (Weinstein *et al.* 2018) | A review of attitudes towards teamworking in an MDT, using the TeamSTEPPS Teamwork Attitude Questionnaire. It examines how to achieve the most effective patient-centred and team-based care through clinical, didactic, leadership, and collaborative training. Not relevant to the question as roles are not explored (if nursing as a leadership or case manager then that could have been included). |
| (Frye 2018) | The use of a pharmacist to arrange Ambulatory Blood Pressure Measurements, review the results, and suggest care—most suggestions were accepted by the Primary Care Provider—although their specific role (GP / NP) is not reported. Only a small number of people (39 over 10 months) were involved. There is limited data on its effectiveness due to the lack of defined roles. In the American context, the study is relevant, but in the UK, treatment protocols follow NICE guidelines, so pharmacy input would not typically be required for medication decisions unless dealing with complex polypharmacy. The question is not fully addressed—the role could be performed by a Healthcare Assistant in the UK. |
| (Fontil *et al.* 2018) | Looking at a protocolled rapid intervention for HTN management, which improved outcomes but provided no comment on who was managing the treatment. Not relevant to the question. |
| (Jindal *et al.* 2018) | Development of a treatment/management app for HTN and T2DM. No stated intention for this to be used as task shifting. No data on effectiveness. Not relevant to the question. |
| (Tu *et al.* 2018) | Study protocol, no data, focused on primary/secondary care collaboration and HTN / T2DM together. Not relevant to the question. |
| (Khan *et al.* 2019) | This is a process development to improve HTN management. An MDT is used but the roles are not discussed. Not relevant to the question. |
| (Rodriguez *et al.* 2019) | Looking at how home-produced clinical data can be managed to prevent overwhelming clinicians and notes with information that is not clinically relevant. Not relevant to the question. |
| (Sadeghi *et al.* 2020) | Helpful on how to achieve medication adherence/medication reconciliation and how to assess education and support needs for patients, but does not address the question. |
| (Moise *et al.* 2020) | A study protocol as part of a push to ensure a HTN diagnosis is based on Ambulatory or Home Blood Pressure Monitoring for greater accuracy. No data. Not relevant to the question. |
| (Biola *et al.* 2020) | Looking at managing severe hypertension, often found in disenfranchised or marginalised groups. A team approach is advised, GP is involved with a ‘patient navigator’ or ‘care coordinator’, but not nurses. Pharmacists were supporting but more to do with managing to costs of medication as many did not have health insurance. Interesting but not relevant to the question. |
| (Margolis *et al.* 2020) | Study protocol. No data. Pharmacist-directed care vs routine care – appears to be GP, with some additional telemonitoring component. Might be useful when completed. |
| (Parra *et al.* 2020) | A review on how best to manage compliance with lifestyle changes and medication adherence for those treated for HTN. Not relevant to the question of who should do the management. |
| (Tobase *et al.* 2020) | Limited information and small-scale study – appears to be looking at various *other* diagnoses made by nurses managing those with HTN – such as overweight / stress / risk of falls etc. No clear suggestion that nurses were making the diagnosis of HTN, nor were the authors suggesting that they should. |
| (Bryant *et al.* 2020) | Telemonitoring with nursing oversight, unable to distinguish the cause of any improved outcomes to the nursing management or the telemonitoring aspect. Trial would need to be focussed on the nursing management alone. Not directly relevant to the question. |
| (Lee *et al.* 2021) | A review of Interprofessional Collaborative Practice (ICP) in Primary Care and how this affects health outcomes for HTN and diabetes. ICP is defined as collaboration between 3 different health professionals, with one person serving as primary carer for diagnosis and treatment initiation. There is no examination of who should hold that role. |
| (Gastens *et al.* 2022) | A protocol for a systematic review examining independent management of hypertension by pharmacists versus usual care. Would be relevant once completed. No data. |
| (Pidugu *et al.* 2022) | An assessment of the potential improvements in HTN control if the WHO HEARTS programme was implemented, focusing on expected enhancements in HTN management and the lives and funds saved by its introduction. Relevant to the necessity of enhancing HTN care but not to the question of who should deliver it. |
| (Malik *et al.* 2022) | A look at the role of the Community Pharmacist in counselling and support for those with HTN. This is an adjunct to the treatment process, but does not look at who makes the diagnosis / starts treatment etc. Not helpful with the question. |
| (Lee *et al.* 2022) | The focus is on managing HTN through remote monitoring. It does not consider who is best equipped to respond to the information, as it assumes that team-based care involves nurses and ‘clinicians’ – implying medical staff – and that decision-making based on remote data is handled by the medical team. It does not address the main question. |
| (Green *et al.* 2022) | Looking at Ambulatory Blood Pressure Monitoring (ABPM) to diagnose HTN, which is gold standard and routinely done in the UK - Not relevant to the proposal – the method used to confirm the diagnosis before treatment would be used by any practitioner who manages HTN – and NICE prefers ABPM so this would be in the protocol / agreed standard of practice. |
| (Costa *et al.* 2022) | The trial was unable to show any improvement in outcomes from pharmacy support for primary care but suggests this is due to limitations in the primary care technology, indicating that the data to support or refute this with certainty is not available. It would be more useful against an increased pharmacy role if the authors were less cautious about their own findings. |
| (Nunes Oliveira *et al.* 2022) | A qualitative study – no review of the roles other than discussion of horizontal working practices and communication issues. Not relevant to the question. |
| (Harris *et al.* 2022) | A look at Train the Trainer (cascade training) in refugee camps for non-communicable disease – inc HTN. Talk about the MDT and team management but no focus on who should do what. |
| (Jindal *et al.* 2022) | Proposal for an IT system to allow task shifting in the management of HTN but not looking specifically at who should be doing this. |
| (Kerry *et al.* 2023) | Considering whether GPS are needed or if all care can be nurse-led -looking at patient complexity when managing six main conditions including hypertension but limited data to pull for HTN only. |
| (Einhorn *et al.* 2023) | Using text messaging to support people in checking their home BP is not relevant to the proposal – if effective, it could be used by any practitioner managing HTN. The proposal is considering who this should be. |
| (Esperat *et al.* 2023) | A review of attitudes towards teamworking in an MDT, using the TeamSTEPPS Teamwork Attitude Questionnaire. This examines how to deliver the most effective patient-centred and team-based care, considering not only the views of the team but also patient health outcomes. Not relevant to the question as roles are not explored in depth – although there is some mention of nursing care management. |
| (Kamal *et al.* 2023) | A team approach to identifying poorly controlled or undiagnosed HTN – No clear role parameters or functions for team members. Limited data available at the time of the report. |
| (Ng *et al.* 2023) | Highlights the lack of clarity about roles and responsibilities in HTN management, but is based on a qualitative study, not exploring what staff or patients would want, which would address the question on a qualitative level. |
| (Hives *et al.* 2023) | An overview of lifestyle changes for pre-hypertension, using pharmacies or community health providers. Not relevant to the question. |
| (Yu *et al.* 2023) | Mainly focused on T2DM and HTN, examining the current poor control of both, and highlighting that lifestyle changes need to be part of routine care – but not clearly answering the question. |
| (Theilmann *et al.* 2023) | A study protocol. No data available yet. Will be relevant once completed, as one control arm involves nurses trained in screening, treatment initiation, lifestyle counselling, and follow-up according to an SOP — with complex cases referred to hospital. The results will support or refute nurses taking on this role when published. |
| (Hysong *et al.* 2023) | Large prospective observational base review of team member construction in primary care. Is awaiting peer review so cannot use the information. |
| (Teo *et al.* 2024) | Care remained GP-led for both the trial and control groups; there was some increased nursing care in the trial group, but it was not possible to determine whether the improved outcomes were due to nurse management or the telemonitoring aspect. The trial would need to focus on nursing management to serve as solid evidence. |
| (Funes Hernandez *et al.* 2024) | A protocol for developing an application to monitor and treat HTN. No actual data is provided and there is no clear plan for this to be part of a task-shifting initiative. Not relevant to the question. |

**References.**

**Alexander A** (2016) Hypertension and Chronic Kidney Disease in Primary Care. https://doi.org/10.15766/mep_2374.

**Barreto M da S, Matsuda LM and Marcon SS** (2016) Factors associated with inadequate blood pressure control in patients of primary care. *Escola Anna Nery - Revista de Enfermagem* 20(1). https://doi.org/10.5935/1414-8145.20160016.

**Biola H, Deyo J, Hayes T, Small L, Chaplin J, Pak-Harvey E, Granger T, Jung L, Fitzgerald L, Crowder C, Patel B, Stillwell T, Eisenson H and Granger B** (2020) Reaching the Hard-to-Reach: Outcomes of the Severe Hypertension Outreach Intervention. *American Journal of Preventive Medicine* 59(5), 725–732. https://doi.org/10.1016/j.amepre.2020.05.030.

**Bryant KB, Liyanage-Don N, Moise N, de Miguel MH, Singer JR, Shim HG, Fuller WS, Gee C, Bona A and Kronish IM** (2020) Implementation of a home blood pressure telemonitoring program to improve hypertension control in a Primary Care clinic. *Journal of General Internal Medicine - Abstracts from the 2020 Annual Meeting of the Society of General Internal Medicine* 35, S656–S657. https://doi.org/10.1007/s11606-020-05890-3.

**Costa S, Biscaia JL, Horta MR, Romano S, Guerreiro J, Heudtlass P, Cary M, Romao M, Rodrigues AT, Miranda A, Martins AP, Bento AS, Pereira. Joao, Mateus C and Helling D** (2022) Real-world effectiveness in hypertension and hyperlipidemia collaborative management between pharmacies and primary care in Portugal: A multicenter quasi-experimental pragmatic controlled trial (usfarmácia®). In *International Pharmaceutical Federation Conference Abstracts Seville* , Vol. 23. International Pharmaceutical Federation (FIP), 297–298. https://doi.org/10.46542/pe.2023.233.262300.

**Dobbins MI, Thomas SA, Melton SLS and Lee S** (2016, June 1) Integrated Care and the Evolution of the Multidisciplinary Team. *Primary Care - Clinics in Office Practice*. W.B. Saunders, 177–190. https://doi.org/10.1016/j.pop.2016.01.003.

**Eads V and Maruzzella G** (2016) The Clinical Nurse as an Agent of Change. *Medsurg Nursing* 25(3), 173–175.

**Einhorn J, Murphy AR, Rogal SS, Suffoletto B, Irizarry T, Rollman BL, Forman DE and Muldoon MF** (2023) Automated Messaging Program to Facilitate Systematic Home Blood Pressure Monitoring: Qualitative Analysis of Provider Interviews. *JMIR Cardio* 7(1). https://doi.org/10.2196/51316.

**Esperat MC, Hust C, Song H, Garcia M and McMurry LJ** (2023) Interprofessional Collaborative Practice: Management of Chronic Disease and Mental Health Issues in Primary Care. *Public Health Reports* 138(1_suppl), 29S-35S. https://doi.org/10.1177/00333549231155469.

**Fontil V, Gupta R, Moise N, Chen E, Guzman D, Mcculloch CE and Bibbins-Domingo K** (2018) Adapting and evaluating a health system intervention from Kaiser Permanente to improve hypertension management and control in a large network of safety-net clinics. *Circulation: Cardiovascular Quality and Outcomes* 11(7). https://doi.org/10.1161/CIRCOUTCOMES.

**Frye A** (2018) Development and implementation of a clinical pharmacist-led ambulatory blood pressure monitoring service in a primary care medical home. In *Journal of the American College of Clinical Pharmacy - 2018 Global Conference on Clinical Pharmacy*, Vol. 1. Wiley, 298–298. https://doi.org/10.1002/jac5.1059.

**Funes Hernandez M, Babakhanian M, Chen TP, Sarraju A, Seninger C, Ravi V, Azizi Z, Tooley J, Chang TI, Lu Y, Downing NL, Rodriguez F, Li RC, Sandhu AT, Turakhia M, Bhalla V and Wang PJ** (2024) Design and Implementation of an Electronic Health Record-Integrated Hypertension Management Application. *Journal of the American Heart Association* 13(2), e030884. https://doi.org/10.1161/JAHA.123.030884.

**Gastens V, Kiszio B, Del Giovane C, Tsuyuki R, Paradis G, Chiolero A and Santschi V** (2022) Pharmacist interventions to improve hypertension management: protocol for a systematic review of randomised controlled trials. *BMJ Open* 12(5), e059399. https://doi.org/10.1136/bmjopen-2021-059399.

**Green BB, Anderson ML, Ehrlich K, Hall YN, Hansell LD, Hsu C, Joseph D, Margolis KL, McClure JB, Munson SA and Thompson MJ** (2022) Blood Pressure Checks for Diagnosing Hypertension: Health Professionals’ Knowledge, Beliefs, and Practices. *Journal of the American Board of Family Medicine* 35(2), 310–319. https://doi.org/10.3122/jabfm.2022.02.210318.

**Harris P, Kirkland R, Masanja S, Le Feuvre P, Montgomery S, Ansbro É, Woodman M and Harris M** (2022) Strengthening the primary care workforce to deliver high-quality care for non-communicable diseases in refugee settings: Lessons learnt from a UNHCR partnership. *BMJ Global Health* 7. https://doi.org/10.1136/bmjgh-2021-007334.

**Hives L, Georgiou RF, Spencer J, Benedetto V, Clegg A, Rutter P, Watkins C, Williams N and Bray EP** (2023) Risk reduction intervention for raised blood pressure (REVERSE): Protocol for a mixed-methods feasibility study. *BMJ Open* 13(5). https://doi.org/10.1136/bmjopen-2023-072225.

**Hong JC, Padula W V, Hollin IL, Hussain T, Dietz KB, Halbert JP, Marsteller JA and Cooper LA** (2017) *Care Management to Reduce Disparities and Control Hypertension in Primary Care. A Cost-effectiveness Analysis*. Retrieved from www.lww-medicalcare.com

**Hussain T, Allen A, Halbert J, Anderson CAM, Boonyasai RT and Cooper LA** (2015) Provider Perspectives on Essential Functions for Care Management in the Collaborative Treatment of Hypertension: The P.A.R.T.N.E.R. Framework. *Journal of General Internal Medicine* 30(4), 454–461. https://doi.org/10.1007/s11606-014-3130-4.

**Hysong SJ, Arredondo K, Lester HF, Sorelle R, Pham T, Oswald FL, Woodard L, Petersen LA, Hamer J and Hughes AM** (2023) Impact of Primary Care Team Configuration on Access and Quality of Care. *MedRxiv*. https://doi.org/10.1101/2023.05.18.23290117.

**Jackson GL, Weinberger M, Kirshner MA, Stechuchak KM, Melnyk SD, Bosworth HB, Coffman CJ, Neelon B, Van Houtven C, Gentry PW, Morris IJ, Rose CM, Taylor JP, May CL, Han B, Wainwright C, Alkon A, Powell L and Edelman D** (2016) Open-label randomized trial of titrated disease management for patients with hypertension: Study design and baseline sample characteristics. *Contemporary Clinical Trials* 50, 5–15. https://doi.org/10.1016/j.cct.2016.07.009.

**Jindal D, Gupta P, Jha D, Ajay VS, Goenka S, Jacob P, Mehrotra K, Perel P, Nyong J, Roy A, Tandon N, Prabhakaran D and Patel V** (2018) Development of mWellcare: an mHealth intervention for integrated management of hypertension and diabetes in low-resource settings. *Global Health Action* 11(1). https://doi.org/10.1080/16549716.2018.1517930.

**Jindal D, Sharma H, Gupta Y, Ajay VS, Roy A, Sharma R, Ali M, Jarhyan P, Gupta P, Srinivasapura Venkateshmurthy N, Ali MK, Narayan KMV, Prabhakaran D, Weber MB, Mohan S, Patel SA and Tandon N** (2022) Improving care for hypertension and diabetes in india by addition of clinical decision support system and task shifting in the national NCD program: I-TREC model of care. *BMC Health Services Research* 22(1). https://doi.org/10.1186/s12913-022-08025-y.

**Kamal M, Chaudhry SA, Vakili V, Haider S, Caplan K, Rughwani N and Baharlou S** (2023) Take the Pressure Off: Team Approach in Optimizing HTN in a Primary Geriatrics Clinic - C187. In *Journal of the American Geriatrics Society - Annual Scientific Meeting. Volume 71*, Vol. 71. Wiley, S249. https://doi.org/10.1111/jgs.18336.

**Kerry TP, Cudahy PGT, Holst HL, Ramsunder A and McGrath NG** (2023) A doctor at a PHC clinic: A ‘must-have’ or ‘nice-to-have’? *South African Medical Journal* 113(1), 24–30. https://doi.org/10.7196/SAMJ.2023.V113I1.16700.

**Khan H, Sadeghi C, Tiemeier A and Bakhai S** (2019) Improving blood pressure control by optimization of guideline directed therapy for hypertension in a Primary Care setting: Quality Improvement Project. *Journal of Hypertension* 37, e188.

**Lalonde L, Goudreau J, Hudon É, Lussier MT, Bareil C, Duhamel F, Lévesque L, Turcotte A, Lalonde G, Montigny M, Rousseau J, Vanier MC, Couture D, Solange Boucher N, Andrée Giguère N, Lise Bertrand N, Marie-France Dubois N, Estelle Lafortune N, Sophie Langlois N, Guèvremont C, Pagé J, Major S, Riano P, Andrée Delorme N, Gauthier L, Heppell J, Couture P, Damestoy N, Desrochers J, Martineau J, Gagnon C and Faubert S** (2014) Development of an interprofessional program for cardiovascular prevention in primary care: A participatory research approach. *SAGE Open Medicine* 2. https://doi.org/10.1177/2050312114522788.

**Lee JK, McCutcheon LRM, Fazel MT, Cooley JH and Slack MK** (2021) Assessment of Interprofessional Collaborative Practices and Outcomes in Adults with Diabetes and Hypertension in Primary Care: A Systematic Review and Meta-analysis. *JAMA Network Open* 4(2). https://doi.org/10.1001/jamanetworkopen.2020.36725.

**Lee NS, Anastos-Wallen R, Chaiyachati KH, Reitz C, Asch DA and Mehta** SJ (2022) Clinician Decisions after Notification of Elevated Blood Pressure Measurements from Patients in a Remote Monitoring Program. *JAMA Network Open* 5(1). https://doi.org/10.1001/jamanetworkopen.2021.43590.

**Malik M, Hussain A, Aslam U, Hashmi A, Vaismoradi M, Hayat K and Jamshed S** (2022) Effectiveness of Community Pharmacy Diabetes and Hypertension Care Program: An Unexplored Opportunity for Community Pharmacists in Pakistan. *Frontiers in Pharmacology* 13. https://doi.org/10.3389/fphar.2022.710617.

**Margolis KL, Crain AL, Bergdall AR, Beran MS, Anderson JP, Solberg LI, O’Connor PJ, Sperl-Hillen JAM, Pawloski PA, Ziegenfuss JY, Rehrauer D, Norton C, Haugen P, Green BB, McKinney Z, Kodet A, Appana D, Sharma R, Trower NK, Williams RA and Crabtree BF** (2020) Design of a pragmatic cluster-randomized trial comparing telehealth care and best practice clinic-based care for uncontrolled high blood pressure. *Contemporary Clinical Trials* 92. https://doi.org/10.1016/j.cct.2020.105939.

**Mino-Leõn D, Reyes-Morales H and Flores-Hernández** S (2015) Effectiveness of involving pharmacists in the process of ambulatory health care to improve drug treatment adherence and disease control. *Journal of Evaluation in Clinical Practice* 21(1), 7–12. https://doi.org/10.1111/jep.12207.

**Moise N, Phillips E, Carter E, Alcantara C, Julian J, Thanataveerat A, Schwartz JE, Ye S, Duran A, Shimbo D and Kronish IM** (2020) Design and study protocol for a cluster randomized trial of a multi-faceted implementation strategy to increase the uptake of the USPSTF hypertension screening recommendations: The EMBRACE study. *Implementation Science* 15(1). https://doi.org/10.1186/s13012-020-01017-8.

**Ng CJ, Tan R, Moose SA, Oka P, Lee CS and Tan NC** (2023) Challenges in managing hypertension in a Singapore Primary Care setting: A qualitative study. *Journal Of Hypertension* 41, e470.

**Nunes Oliveira C, Galvão Oliveira M, Wildes Amorim W, Nicolaevna Kochergin C, Mistro S, de Medeiros DS, Oliveira Silva K, Moraes Bezerra V, Honorato dos Santos de Carvalho VC, Bispo Júnior JP, Louzado JA, Lopes Cortes M and Arruda Soares D** (2022) Physicians’ and nurses’ perspective on chronic disease care practices in Primary Health Care in Brazil: a qualitative study. *BMC Health Services Research* 22(1). https://doi.org/10.1186/s12913-022-08078-z.

**O’Donnell AJ, Bogner HR, Cronholm PF, Kellom K, Miller-Day M, de Vries McClintock HF, Kaye EM and Gabbay R** (2016) Stakeholder perspectives on changes in hypertension care under the patient-centered medical home. *Preventing Chronic Disease* 13(2). https://doi.org/10.5888/pcd13.150383.

**Parra DI, Trapero Gimeno I, Sánchez Rodríguez JM, Rodríguez Corredor LC, Hernández Vargas JA, López Romero LA, Garciá López FJ, Escudero-Gómez C, Trujillo-Cáceres SJ, Serrano-Gallardo P and Vera-Cala LM** (2020) Individual interventions to improve adherence to pharmaceutical treatment, diet and physical activity among adults with primary hypertension. A systematic review protocol. *BMJ Open* 10(12). https://doi.org/10.1136/bmjopen-2020-037920.

**Pidugu A, Pickersgill S, Watkins D, Husain J, Kostova D, Farrell M, Haider M, Jubayer S, Tarannum R, Bhuiyan M, Moran AE and Choudhury S** (2022) National health and budget impact of implementing the WHO HEARTS hypertension control program in Bangladesh. *The Lancet Global Health* 10, S23. https://doi.org/10.1016/s2214-109x(22)00152-8.

**Piper MA, Evans C V, Burda BU, Margolis KL, O’connor E, Smith N, Webber E, Perdue LA, Bigler KD and Whitlock EP** (2014) *Screening for High Blood Pressure in Adults: A Systematic Evidence Review for the U.S. Preventive Services Task Force. Evidence Synthesis No. 121*. Retrieved from www.ahrq.gov

**Rodriguez S, Hwang K and Wang J** (2019) Connecting home-based self-monitoring of blood pressure data into electronic health records for hypertension care: A qualitative inquiry with primary care providers. *JMIR Formative Research* 3(2). https://doi.org/10.2196/10388.

**Sadeghi C, Khan HA, Gudleski G, Reynolds JL and Bakhai SY** (2020) Multifaceted strategies to improve blood pressure control in a primary care clinic: A quality improvement project. *International Journal of Cardiology: Hypertension* 7. https://doi.org/10.1016/j.ijchy.2020.100060.

**Santschi V, Wuerzner G, Chiolero A, Burnand B, Schaller P, Cloutier L, Paradis G and Burnier M** (2017) Team-based care for improving hypertension management among outpatients (TBC-HTA): Study protocol for a pragmatic randomized controlled trial. *BMC Cardiovascular Disorders* 17(1). https://doi.org/10.1186/s12872-017-0472-y.

**Skar P, Young L and Gordon C** (2015, October 1) Changes in blood pressure among users of lay health worker or volunteer operated community-based blood pressure programs over time: a systematic review protocol. *JBI Database of Systematic Reviews and Implementation Reports*. 30–40. https://doi.org/10.11124/jbisrir-2015-1927.

**Teo VHY, Teo SH, Burkill SM, Wang Y, Chew EAL, Ng DWL, Tang WE and Koh GCH** (2024) Effects of technology-enabled blood pressure monitoring in primary care: A quasi-experimental trial. *Journal of Telemedicine and Telecare* 30(1), 121–130. https://doi.org/10.1177/1357633X211031780.

**Theilmann M, Ginindza N, Myeni J, Dlamini S, Cindzi BT, Dlamini D, Dlamini TL, Greve M, Harkare HV, Hleta M, Khumalo P, Kolbe LM, Lewin S, Marowa LR, Masuku S, Mavuso D, Molemans M, Ntshalintshali N, Nxumalo N, Osetinsky B, Pell C, Reis R, Shabalala F, Simelane BR, Stehr L, Tediosi F, van Leth F, De Neve JW, Bärnighausen T and Geldsetzer P** (2023) Strengthening primary care for diabetes and hypertension in Eswatini: study protocol for a nationwide cluster-randomized controlled trial. *Trials* 24(1). https://doi.org/10.1186/s13063-023-07096-4.

**Tobase L, Bella ECPM, Polastri TF, Tomazini EAS, deSouza DR, Peres HHC, Nogueira GL and Carvalho CF** (2020) Nursing diagnoses in people with hypertension in primary health care. MP-71. In *Journal of clinical hypertension - Abstracts of the XXVII Annual Meeting of the Brazilian Society of Hypertension*. NLM (Medline), 773. https://doi.org/10.1111/jch.13745.

**Tomiak E, Chlabicz S, Mizgała E, Lukas W, Panasiuk L, Drzastwa W and Jankowska-Zduńczyk A** (2016) Prevention of cardiovascular disease in a rural general practice. *Annals of Agricultural and Environmental Medicine* 23(4), 553–558. https://doi.org/10.5604/12321966.1226845.

**Tu Q, Xiao LD, Ullah S, Fuller J and Du H** (2018) Hypertension management for community-dwelling older people with diabetes in Nanchang, China: Study protocol for a cluster randomized controlled trial. *Trials* 19(1). https://doi.org/10.1186/s13063-018-2766-5.

**Weinstein AR, Dolce MC, Koster M, Parikh R, Hamlyn E, McNamara EA, Carlson A and DiVall M V.** (2018) Integration of systematic clinical interprofessional training in a student-faculty collaborative primary care practice. *Journal of Interprofessional Care* 32(1), 104–107. https://doi.org/10.1080/13561820.2017.1355296.

**Yu EYT, Wan EYF, Chan KHY, Wong CKH, Kwok RLP, Fong DYT and Lam CLK** (2015) Evaluation of the quality of care of a multi-disciplinary Risk Factor Assessment and Management Programme for Hypertension (RAMP-HT) Service organization, utilization, and delivery of care. *BMC Family Practice* 16(1). https://doi.org/10.1186/s12875-015-0291-0.

**Yu X, Li YT, Cheng H, Zhu S, Hu XJ, Wang JJ, Mohammed BH, Xie YJ, Hernandez J, Wu HF and Wang HHX** (2023) Longitudinal changes in blood pressure and fasting plasma glucose among 5,398 primary care patients with concomitant hypertension and diabetes: An observational study and implications for community-based cardiovascular prevention. *Frontiers in Cardiovascular Medicine* 10. https://doi.org/10.3389/fcvm.2023.1120543.

.
