# Supplementary material for: Practice nurse management of hypertension in primary care: a scoping review
Source: Prim Health Care Res Dev. 2026 Aug 11;27:e89. doi: 10.1017/S1463423626101455 (PMC13740167; doi:10.1017/S1463423626101455)
Supplement: Supplementary file 3 [file S1463423626101455sup003.docx]

**Appendix 3**

**Scoping Review – Summary of Evidence**

| Study | Country | Type of  Evidence | Numbers | Summary of outcomes | Are roles / team structure defined? | Independent  Practice supported? | Does this answer the proposal? | Comments | Evidence  Suggests |
| --- | --- | --- | --- | --- | --- | --- | --- | --- | --- |
| (Maheux *et al.* 2014) | Canada | Qualitative | 186 Graduates in Family Medicine | >70% of Drs are open to collaboration / shared decision making with Nurse Clinicians– but limited clinical decision-making ability witnessed. | Yes - the phrase ‘Nurse Clinician’ would equate to a Nurse Practitioner | No – 68.8% had never seen a nurse initiate treatment | No | The study was looking at what graduates had witnessed/expected, not what they would agree with, or what was happening on the ground. | There may be resistance to increasing PN or pharmacist roles from the medical profession. |
| O’Neill et al., 2014 | United States | Non-randomised retrospective comparison | 126 patients divided into two cohorts – PN and GP care or PN and CP care | CP/PN care patients had moderate improvements in systolic BP over GP care | Yes – the term ‘Registered Nurse Case Manager’ is an RN and not an ANP | Yes – for the CP. The PN is a case manager and data collector only. | Yes – The collaboration between RN and CP was found to be as effective as GP | Low numbers. The PN is the ‘case manager’ in both cohorts – but all clinical decisions are at the CP/GP level | PN as ‘case manager’ with CP as medication manager is slightly more effective than GP |
| Deales et al., 2014 | Italy | Cluster randomised trial | 920 patients enrolled, 726 in final analysis | PN as care managers supporting GPs had significantly better outcomes than GPs alone, with at least one clinical target met in 39% of trial patients compared to 25% for usual GP care. | Yes – the case manager is an RN and not an ANP | No – the study is looking at a disease and care management approach vs usual care – not who provides the care. | Partly - not for independence, but good evidence for care or case management by PN, although low numbers. | The study was looking at improvements in multiple cardiovascular risk factors, not just HTN. Data for HTN suggests 35% of patients in the trial met the target compared to 19% for GP care. | The PN, as ‘case manager’ and educator, has strong support. |
| Martínez-González et al., 2014 | Switzerland | Systematic review and Meta-Analysis of RCTs on Nurse vs Physician care for multiple clinical conditions (Table 1) | 11 RCTs (N=30,247) but only 5 trials looking at HTN specifically, N=1,344 | Nursing care resulted in a greater reduction in Systolic BP, with no difference in diastolic BP. Of 32 clinical parameters for cardiovascular risk, under one-fifth favoured nurse-led care, 25 showed no difference. | Yes – the nurses were PN with no additional training in most trials, but for HTN specifically, they are referred to as Nurse Practitioners only. It is noted that nurses' roles and experience required to substitute for GPs needs further research. | Yes – Nurse Practitioners are better at managing HTN, and trained PN are as good as GPs in managing T2DM, Lipids, Lung function or CKD | Yes – One trial reports full clinical autonomy, the rest report varying levels, but most have independent prescribing of treatment. Outcomes in Fig 2 | While there is no evidence that nurse-led care improves outcomes generally, this provides evidence that their care (based on outcomes) is equal to a GP. Most studies small (<200 patients) with high risk of bias (half had >20% attrition). | Supportive of NP led care for HTN, including the initiation and titration of medication, but also positive about PN independent practice. |
| Allen et al., 2014 | United States | Randomised control trial. COACH study – NP working with community health worker vs Physician care. | 525 patients enrolled, | Patients streamed into the NP / Community health worker team vs the enhanced GP. The NP was the case coordinator. At one year, the NP patients had significant overall improvement in all parameters, staffing costs were lower, but investigations and medication costs were higher. | Not clearly. The term NP has no definition, but there is the implication of independent prescribing, which would suggest postgraduate education | Yes – for Nurse Practitioners, not PN | No, as the care was done by NP’s who are independent practitioners | The increased costs noted were proposed to be the result of increased medication, which is why the BP was better managed, and more frequent testing in line with guidance, which was not done by the GP cohort. | This would support a PN and NP team in the management of HTN. |
| Rumball-Smith et al., 2014 | Canada and New Zealand | A questionnaire looking at the availability of services when increased numbers of ‘non-physicians’ are part of the primary care team. | 352 primary care practices.  184 from Canada and 168 from New Zealand, with 330 in the final analysis. | A review of primary care practices using a questionnaire, to see if they are more likely to provide dedicated chronic condition clinics if they have a broader variety of professions employed in the practice, which it found they are. | No | No - this was not part of the study. | No -but it makes the point that the optimal primary care team has not been defined, and this needs further investigation (p.7) | Tries to distinguish between ‘interdisciplinary’ and ‘multidisciplinary’ in the team approach. There is no analysis of data to support a particular non-physician speciality as improving care of chronic disease. There is no review of outcomes to see if they are improved by increased access. | Supports team care as improving access, but no guidelines on what that should be or who is doing what in the team.  Co-located MDT’s improve capacity to provide broad, specialised and preventative care for chronic disease (LTC’s) |
| Proia et al., 2014 | United States | Systematic review. It is noted the heterogeneity in the various studies prevented a formal meta-analysis. | 52 studies were included in the review. No total number of patients given. 28 interventions used RN’s, 15 used Pharmacists and 5 included both. | A review of team-based care for HTN. The team were supportive of the GP’s care, not a replacement for it. The main additional team components were pharmacists and RN’s. There is strong evidence that team care is effective in improving BP outcomes | Not clearly, but no pharmacist or nurse was noted to be a prescriber. | No – this was not part of the study | Partly, the addition of Nurses in particular, and nurses with pharmacists as part of the team, was shown to be most beneficial over any other specialities, and independent medication management by them was the most effective at HTN control. | Outside of the clinical improvement, team-based care is found to be more cost-effective. Team-based care improves outcomes, improves patient satisfaction and reduces per capita costs (page 11) | Team-based care is found to result in substantial improvements in HTN management**.** Independent medication changes, or recommendations for changes to the GP, were found to be more effective than lifestyle support only with the GP in charge of the medication regimen. |
| Fortuna et al., 2015 | United States | Intervention trial | 13,404 patient visits over a 3-year and five-month period. No total patient numbers included. Noted, 134 patients were intensely monitored, which was around 10% of the total so the estimate of around 1,300 | A review of team-based care for HTN. A pharmacist and RN were used for counselling, medication titration, BP checks and support under the direction of a GP. BP control improved from 51.6% to 67.4% post-intervention | Yes | Partly – Pharmacists provided advice and also titrated medication when required under a protocol. The PN was used as a case manager, ensuring compliance and escalating resistant HTN cases. | Partly – it shows how PN and Pharmacists' management can be more effective than GP, particularly for high-risk patients. | High-risk patients went from <40% in range to 60% in range during the intervention, but dropped back post-intervention. | They were unable to review whether the PN or the pharmacist interventions had the most effect, but both together showed improvements in HTN management. There were no NP’s or PA’s on site. |
| Carrington & Stewart, 2015 | Australia | Observational study | 530 self-selected then screened into risk levels for care planning. | A review of outcomes from a nurse-led clinic for CVD. While the GP review was shown to improve BP, the nurse-led clinic improved absolute CVD risk by all parameters. | No – there is no clear description of the training that the nurse involved in the intervention was given. | No - there is no description of medication titration, just lifestyle/engagement/support and referral. Medication titration would be needed for independent practice. | Partly – it shows how PN management of high-risk patients can lead to improvement but only as counselling/advice/lifestyle change support. |  | Lifestyle changes reduce risk, and PN support can help achieve this. |
| Hussain et al., 2015 | United States | Qualitative analysis | 39 GP’s / NP from six practices | A review of a framework for care management tasks for those who are supporting GPs looking after people with chronic diseases. The PARTNER checklist was found to support collaboration | No - the phrase ‘care manager’ is used, but the qualification/registration of that person is not discussed. | No | No |  | A way of defining the role of a care manager tasked with supporting patients in managing their HTN. |
| Weinstein & Rucker, 2016 | United States | Intervention  Trial | 4021 patients on the registry. | A review of a ‘Treat to Target’ team approach to HTN. PN managed routine HTN, and a specially trained PN ran a special ‘Treat to Target’ programme using motivational interviewing and education. BP control went from 40% to 62%. | Yes. ANPs are noted to be doing other tasks in the surgery, and all nursing support is noted to be RN / PN. | Yes | Yes  RN’s were managing even complex and resistant cases independently using protocols. This involved all aspects of care including medication and follow up. | The focus on the HTN management appointments led to delays in appointments for non-HTN patients despite an additional RN being employed. | RN’s can fully manage even resistant HTN, including medications, in an area of high social mix and deprivation. |
| Price-Haywood et al., 2017 | United States | Retrospective Observational Study | Patients with HTN  N=1111  Treated by CP  N=236 | A review of collaborative care for HTN patients involving GP and CP vs GP care alone. The median time to achieve control was longer for the collaborative care. | Yes – and protocol for HTN management/medication is provided | No – this was a GP vs collaborative care model. | Partly, this study provides evidence that collaborative care with a CP increases time to BP management, but does not discuss PN’s. | The cohort treated by the CP had higher rates of comorbidities compared to the GP, had poor disease control before referral, and also had more reviews by the GP compared to GP management alone. | The improvements in HTN management expected by team working may not be achievable if the cohort of those left to be treated is restricted to resistant or complex cases, although the RN cohort in Weinstein & Rucker (2016) were also complex but showed improvement. |
| Matthys et al., 2017 | Belgium | Systematic Review of peer-reviewed reviews. | 11 Systematic Reviews used, which include a total of 285 primary studies containing 173 RCTs. For HTN, see Tables 3+4. Two Systematic Reviews, Health Quality Ontario (2013) and Shaw et al (2014), total N=13,165. | An evidence synthesis of systematic reviews looking at PN and GP collaboration on LTCs. BP control was exclusively reported as improved in three reviews; other outcomes were mixed. | No – there is an attempt to, but acknowledgement that the original articles have not provided clarity. | No – looking at collaborative working. Some outcomes were worse with the collaboration. The exact meaning of collaboration (independent with support or under the direction of?) is not discussed. | Partly – this is supporting the specific proposal around HTN, but is not supportive of team care in general. | Most reviews conclude that nursing care has added value, although most studies were done by nurses; there needs to be clear roles, tasks, and responsibilities for collaboration to work. | Blood pressure was the only patient outcome exclusively reported in two reviews (two with meta-analysis). Five reviews showed improved patient satisfaction, and four reviews showed decreased hospitalisation, with one review suggesting hospitalisation was increased. Low to moderate quality evidence. |
| Ciapponi et al., 2017 | Cochrane Library - International | Overview of systematic reviews | 51 Systematic reviews.  Two studies were identified specifically for HTN. Brownstein et al (2007) with 14 studies  N=61,246 (Table 1) and Marinez-Gonzalez et al (2014) with 11 studies N=15,999 (Table 1) | Overview of systematic reviews looking at care delivery arrangements and patient outcomes in low-income countries. Lay or community health workers improved behaviour, BP and reduced mortality for those with HTN (14 studies). Physician-nurse substitution showed improved BP for those with HTN but generally had no other positive or detrimental effect (11 studies) | No | Yes | Yes – concerns about PN managing HTN when lay or unregistered members of the health care team can achieve improvements on GP care are indefensible. | There was no moderate or high certainty evidence of undesirable effects from the multiple changes reviewed. There was moderate to high certainty evidence supporting lay or community health workers managing people with HTN, and nurses substituting for physicians in HTN, heart failure, diabetes, asthma, Parkinson’s and incontinence – with no evidence of negative outcomes. | The 14 studies on lay health workers were done in high-income settings (ie, the improved outcomes were not on the background of underfunded care) and would suggest the use of HCAs in this role would be justified. This is not the same Martinez-Gonzalez et al (2014) above but ‘Integrated care programmes for adults with chronic conditions: a meta-review’ |
| Lukewich et al., 2018 | Canada | Cross-sectional survey | Completed questionnaires from 26 community located practices – Family Health Teams (FHT, n=21)) and Community Health Centres (CHC, n=4) | A review of primary care teams, their composition with regard to different professionals in them, the various nursing roles and chronic disease management. NP and PN are the most common non-physician providers, and their roles overlap. | Nursing roles are clearly defined in Table 1. A NP is an ANP. Care responsibilities are recorded in Table 3.  It is not made clear the level of independent practice for PN, and the overlap and confusion about roles is acknowledged | This is not what the paper is looking at. | No – it is looking at the composition of teams in primary care, with some definition of roles but not deciding on what can or should be done by each role. | In this Canadian survey, PN’s are noted to have less education and have a reduced scope of practice compared to RN’s. This is not the case in the UK, where all new PN now have post-graduate courses allowing for independent speciality management, screening and immunisations. | Nursing roles vary between different sites. It is not clear if a GP surgery in the UK would be an FHT or a CHC, but FHTs all had systematic management of hypertension. Nursing roles are poorly defined and often overlapping, meaning their efficient use and full scope of practice may not be realised. |
| Gorina et al., 2018 | Spain | Systematic review without meta-analysis | 20 studies  N=15,439 | A review of the evidence of the effectiveness of educational interventions used by nurses to improve outcomes in chronic disease | Nursing is mentioned as a profession, but no specifics on the type of nurses are given | Only as educators, the paper is not looking at the clinical management | Partly, it is looking at the evidence for the best way to educate patients on their chronic disease. |  | Group, individual face-to-face or telephone support all show statistically significant changes. |
| Derington et al., 2019 | United States | Use of Cost-effectiveness analysis to assess which forms of team-based care has better outcomes and savings from intensive BP management. | Using the SPRINT trial of 2015 to support intensive BP management.  References Mills et al (2018) review of team-based care N=55,920 | A review of the benefits of team-based care (TBC), which was more effective at lowering systolic BP with non-physician prescribing than with physician prescribing. | Nursing and NP are mentioned, but there are no studies reported that compare the two doing the same job within the team. The legal parameters for the scope of practice are mentioned. | Partly, as part of a team, the composition of which has not been properly researched. | Yes – team-based care is cost-effective compared to usual care | Provides a thorough review of the evidence base for intensive BP control. Provides support for the cost-effectiveness of team-based care, both in the cost of the team but also on the long-term savings to the health system from the improved clinical outcomes and reduced morbidity and mortality. | 100 trials with >55,000 patients suggest that a multilevel, multicomponent approach, often with non-physician care and medication changes, was the most effective arrangement. Pharmacists and RN were the two most studied care managers and were both more effective than usual care. Nursing care was more effective when titrating the medication themselves than when not. |
| Schor et al., 2019 | Israel | Cross-sectional survey | 516 Clinics responded to the survey | A survey looking at health education and promotion in primary care. Collaboration (GP and PN) or Teamwork (PN / dietician / social worker and GP) were found to be more effective than GP-led care. | There are no bands of nursing mentioned – assuming RN / PN level | No – the paper is looking at health education rates, not clinical management or outcomes. | Yes – as care manager role with GP. |  | All health outcomes were significantly improved in the Teamworking groups compared to GP care alone. Collaboration and teamwork outcomes were similar, with increased investment and management required for teamworking compared to collaboration. |
| Girvin, 2019 | UK | Journal Article |  | Advice on CP running a HTN clinic | Not relevant | For CP – but the format is expandable to others | Yes | Sets out testing etc required to manage HTN independently. Venepuncture and ECG recording could be done by the PN themselves. | Practical advice on legal, clinical and practical preparations for a pharmacist to run an HTN clinic. It is noted they will have more time with the patients than GP’s. |
| Lee et al., 2019 | United States | Intervention trial | 4,762 patients with HTN diagnosis | The implementation of a HTN care pathway. Control (last noted BP in range) improved by 11.6% over six months. The use of Lean thinking (from production lines) was applied to the processes to improve efficiency. | No. NP’s are mentioned as interchangeable with GP or PA, but this is not relevant to the study, which is looking at the application of change to the system. | RN is used to treat the target with up to 3 meds using GP-delegated orders. | Yes, but caution is not the focus of the study, which is the implementation of the pathway. | Improvements were achieved without additional funding.  Improving BP control in 628 people from 67% to >80% would prevent 9 strokes and 6 MIs, of which 5 would die. It achieved 78.2% improvement by the end of the study. | It looked to make HTN management easier, better, faster and cheaper. Cost savings come from using lower-cost team members and preventing hospitalisation and disability. The efficiency comes from designing an integrated system, avoiding top-down control. |
| Ödesjö et al., 2019 | Sweden | Retrospective cohort study – cross-sectional register-based review | 88,945 patients with HTN | Only 48% of patients with HTN are controlled. Increased visits to nurses or management of HTN by nurses improved BP control, but GP reviews did not. | No, and it is mentioned that only GPs can prescribe anti-hypertensive medication in Sweden. | Yes – nursing care is superior to GP care. It is proposed that this is due to strict adherence to a treatment protocol. | Yes - The intervention was technically teams-based care vs GP care as only the GP can prescribe. | Patients with nurse-based care had a 10% higher chance of achieving HTN control, which increased with the number of visits they had. | Patients had more contact and were started on more medication with the PN care. From the description, the Nurse is the care coordinator, and the improved outcomes are found in other studies where nurses take on this role. |
| Marco-Moreno et al., 2020 | Spain | Qualitative  Focus Group | 8 | The views of GP’s involved in the ADAMPA trial, in which patients monitor their BP and titrate their own medication to target. Early outcomes seem positive. | Not relevant | No | Partly - in so far as it shows the level of risk is low, and concerns about nurses taking on a GP role are unfounded | Self-titration of medication is common for diabetics and conditions such as asthma and COPD, so the concept is not new. | It acknowledges the conflict between the GP and the PN regarding the competency of the nurse. Do GP’s trust patients with their medication more than nurses? |
| Perris, 2020 | United States | Journal Article |  | The use of standardised procedures allows RN / PN to give independent care for patients. The discussion on agreed medication would meet the criteria for a PGD. | Yes – this is a review of a specific project allowing PN to undertake independent clinical reviews, form a diagnosis and issue medication. | Yes, but working to strict protocols about what they can and cannot do | Yes |  | The use of protocols, based on NICE guidelines, and PGD medications -limiting the types and dosage of medication issued- could be applied to PN HTN management. |
| John et al., 2020 | United States – international studies used. | Systematic review and Meta-analysis of RCT / Non-RCT | 85 trials covering 60,617 patients | A review of chronic disease management outcomes (clinical and financial) with team care vs GP care. While team care was significantly better in chronic disease management generally, reductions in BP overall were not statistically significant; the care costs more, and the improvements may not be cost-effective. | No - It is based on the Primary Care Medical Home format so assume that there are protocols for staff roles/mix and ratios. | No, not what it was intending to do | Yes – partly relevant to the question as it is not supportive of team care involving nurses, within the Primary Care Medical Home structure. | The high heterogeneity of outcomes used in the pooled evidence, with some low-grade evidence in the numbers, may limit conclusions (p.17). | Conclusions support team-based care on the basis of outcomes for multiple medical conditions, apart from HTN. The GP is seen as the care coordinator, who can allocate less complex patients to other members of the team. It acknowledges that while initial costs may be higher, improved outcomes may result in overall cost effectiveness. |
| Lin et al., 2020 | United States | Intervention trial | HTN managed in a nurse clinic, 717 patients having 1243 reviews. | Establishing a nurse-run HTN clinic. Issues with staffing, significant no-show rates (50%) that required additional patient prompting and support, BP control actually fell at the start, but did improve towards the end of the trial but only a few percentage points above where they started | The phrase ‘nurse’ is used without elaboration. | No | Partly – very little detail, but the nurse-led clinic showed little improvement. While not discussed, it is assumed there is medical oversight for the provision of medication. | The percentage of patients with controlled HTN initially declined before a slight improvement from the baseline. | The significant no-show rates for the nurse clinic are interesting and may indicate poor public acceptance for a nurse-run clinic or a perceived lack of importance for the condition of HTN. |
| Van Grondelle et al., 2022 | Netherlands | Qualitative study of healthcare providers | 9 GPs, 6 PN and 5 secondary care clinicians | A review of healthcare providers' opinions on the reasons for poor medication compliance. | N/A | Not directly addressed | The study is not relevant to the question |  | Patient engagement for a symptom-free disease is seen as a problem, which is why personal engagement from clinicians is seen as important and suggests this is why many studies show improved outcomes with extra time and contact being more important than clinical skill levels. |
| Nunes Oliveira et al., 2022 | Brazil | Qualitative study of healthcare providers | 5 GP and 18 RN | A review of primary care providers' opinions on providing care for people with chronic conditions | No | No | No |  | The use of the term ‘Non-communicable disease’ could be used to identify a remit for a nursing speciality. The emphasis is on horizontal, rather than hierarchical, team care. |
| Stephen, Halcomb, Batterham, McInnes, et al., 2023 | Australia | Cluster randomised control trial. | 65 patients, 30 in the intervention and 35 in the control. | A pilot trial of a PN intervention to improve BP. Only looking at support for lifestyle changes, the GP still initiates and manages any medication. At 12 months, the intervention group had a mean SBP 11.3 mmHg lower than the control. | Yes | No | Yes – improvements were demonstrated with PN involvement. |  | The nurse is the care coordinator and lifestyle support only, and shows nurse support ‘adds value’. This was a pilot trial of the intervention, and outcomes are not always reported, as it is the process that is under review, so the data were not the focus of the study and should be viewed with caution. |
| Weise et al., 2023 | Germany | Qualitative  Semi-structured interviews with patients | 17 | A review of patient’s opinions and perceptions of PN led consultations and medication dose adjustment. Patients were open to PN led care, felt they were more accessible, but there were concerns about them making medication changes. | Yes | It suggests there may be patient resistance. | Yes | See Figure 1. Some patients were supportive of management of multiple LTC meds, and for the provision of antibiotics and analgesics. | Germany has very limited experience of PN’s, with a history of single and unsupported GP’s. This small study suggests there would be limited opposition to PN taking over the management of HTN, including dose adjustments, but this did not look at starting medication. |
| Hellgren et al., 2023 | Sweden | Clinical management review using Semi-structured interviews | 76 Primary health centres – half GP and half PN responders | A review of how the diagnosis of HTN was made, what tests were done, and what lifestyle questions were asked. It found that European guidelines are not closely followed. | Yes | No - not the purpose of the study | Partly – it shows HTN is being managed by PN in Europe but that the care may not be gold standard clinically, but there is good lifestyle change support. |  | 82% of HTN cases were managed by nurses, but poor ongoing care was demonstrated (lack of follow up bloods/urine tests). Lifestyle changes were supported in >96%. |
| Stephen, Halcomb, Batterham, & Zwar, 2023 | Australia | Qualitative.  Review of clinical teams involved in the Impress Study.  Semi-structured interviews | Three GP’s, five nurses and six patients | A review of clinicians in the Impress study (Stephen, Halcomb, Batterham, McInnes, et al., 2023). All those interviewed were positive about the trial, and felt that HTN management was an important aspect of PC | No | No | Yes – for PN as case manager and support as per the original trial |  | The need for change was agreed upon, and the change proposed by the trial was seen positively. This small study suggests good support by clinicians for new processes, and general agreement that the current system is not working. |
| Martínez-Mardones et al., 2023 | Chile | Cluster randomised control trial. | 324 patients divided into an intervention and control group | A review of pharmacist led medication reviews and follow up for cardiovascular disease including HTN. It was found the reviews improved medication adherence and reduced cardiovascular risk (Fig 2) | Yes | Yes – for pharmacists but arguably in their own scope of practice | Yes |  | While not screening or making the diagnosis, the pharmacists were able to order and review bloods, and record their own clinical findings. Rates of GP acceptance of the pharmacist recommendations >90% are seen as unusual. |
| Yu et al., 2023 | Hong Kong | Prospective cohort study | 212,707 patients from 73 clinics divided into intervention and control groups, followed up over 5 years | A review of the 5 year outcomes for patients with simple hypertension managed by nurses working under a protocol vs usual care. The intervention showed an 8% risk reduction in cardiovascular disease and 10% risk reduction in all-cause mortality. | Not in this paper but in the published supplement. All medication was initiated and managed by the GP. | Yes – under protocol but not for medication | Yes – the nurse is the ‘care manager’ | The number needed to treat to prevent 1 cardiovascular disease event (stroke or heart attack) was 16, for all-cause mortality this was 17. While there were increased outpatient attendances, there was lower hospital-based health care use. Multiple levels of potential bias are noted in the Limitations section. | A large and broad study follow up, showing how improved primary care can reduce secondary care use and improve patient outcomes. These improvements come from increased prescriptions of anti-HTN medications as part of a treatment to target protocol (Table 1). |
| Stojnić et al., 2023) | Slovenia | Qualitative | 48 health care professionals including GP’s, RN’s and PN’s | A review of the perceptions of the phrase ‘integrated care’ for health care professionals. They see collaboration as important, and the nurses as the link between the GP and the patient. | Not clearly. Use of the term practice nurse, registered nurse, community nurse and registered nurse in health education are all listed. | No | Partly | Use of the phrase non-communicable disease to cover diabetes and cardiovascular disease. The role of PN is not know in Slovenia, but this review is supportive of RN’s being part of the primary care team. | Lay support for HTN is supported, as are pharmacy technicians over pharmacists for medication support. GP’s were concerned about fully independent nursing practice such as NP’s. |
| Anand, 2024 | United States | Quality improvement project | 48 patients, self-selecting | Team based intervention for HTN management. RN led with NP or GP medication titration, | Yes | Yes – under protocol but not for medication | Partly – the nurse is the ‘care manager’ | This not only looks at HTN outcomes but patient knowledge of the condition, which is likely to improve lifestyle changes. | Low numbers but supportive of PN and NP collaborative care. |

The abbreviation ‘GP’ is used to denote someone with a medical degree who has qualified in family medicine / primary care.

The abbreviation ‘PN’ is used to denote a Practice Nurse, or any person registered as a Nurse in that country, working in primary care, that does not have additional qualifications to allow for independent practice or hold prescribing rights, but can have some postgraduate training for their role.

The abbreviation ‘NP’ is used to denote any registered nurse who has additional qualifications to allow for independent practice and holds independent prescribing rights.

The abbreviation ‘CP’ is used to denote a Clinical Pharmacist, or a person registered as a pharmacist in that country that has additional independent prescribing rights.

The abbreviation ‘RN’ is used to denote a Registered Nurse.

The abbreviation ‘CVD’ is used to refer to cardiovascular disease.

The abbreviation ‘HTN’ is used to refer to hypertension.

**Bibliography.**

**Allen JK, Dennison Himmelfarb CR, Szanton SL and Frick KD** (2014) Cost-effectiveness of nurse practitioner/community health worker care to reduce cardiovascular health disparities. *Journal of Cardiovascular Nursing* 29(4), 308–314. https://doi.org/10.1097/JCN.0b013e3182945243.

**Anand V** (2024) Team-Based Approach in Hypertension Management: A Quality Improvement Project. *Journal of Nursing Care Quality* 39(1), 76–83. https://doi.org/10.1097/NCQ.0000000000000726.

**Carrington MJ and Stewart S** (2015) Cardiovascular disease prevention via a nurse-facilitated intervention clinic in a regional setting: The Protecting Healthy Hearts Program. *European Journal of Cardiovascular Nursing* 14(4), 352–361. https://doi.org/10.1177/1474515114537022.

**Ciapponi A, Lewin S, Herrera CA, Opiyo N, Pantoja T, Paulsen E, Rada G, Wiysonge CS, Bastías G, Dudley L, Flottorp S, Gagnon MP, Garcia Marti S, Glenton C, Okwundu CI, Peñaloza B, Suleman F and Oxman AD** (2017) *Delivery arrangements for health systems in low-income countries: An overview of systematic reviews* *Cochrane Database of Systematic Reviews*, Vol. 2017. John Wiley and Sons Ltd.

**Deales A, Fratini M, Romano S, Rappelli A, Penco M, Perna GP, Beccaceci G, Borgia R, Palumbo W, Magi M, Vespasiani G, Bronzini M, Musilli A, Nocciolini M, Mezzetti A and Manzoli L** (2014) Care manager to control cardiovascular risk factors in primary care: The Raffaello cluster randomized trial. *Nutrition, Metabolism and Cardiovascular Diseases* 24(5), 563–571. https://doi.org/10.1016/j.numecd.2013.11.008.

**Derington CG, King JB, Bryant KB, McGee BT, Moran AE, Weintraub WS, Bellows BK and Bress AP** (2019, December 1) Cost-Effectiveness and Challenges of Implementing Intensive Blood Pressure Goals and Team-Based Care. *Current Hypertension Reports*. Current Medicine Group LLC 1. https://doi.org/10.1007/s11906-019-0996-x.

**Fortuna RJ, Nagel AK, Rose E, McCann R, Teeters JC, Quigley DD, Bisognano JD, Legette-Sobers S, Liu C and Rocco TA** (2015) Effectiveness of a multidisciplinary intervention to improve hypertension control in an urban underserved practice. *Journal of the American Society of Hypertension* 9(12), 966–974. https://doi.org/10.1016/j.jash.2015.10.004.

**Girvin B** (2019) Primary care hypertension clinics: tips for pharmacist prescribers. *Prescriber* 30(6), 21–24. https://doi.org/10.1002/psb.1768.

**Gorina M, Limonero JT and Álvarez M** (2018) Effectiveness of primary healthcare educational interventions undertaken by nurses to improve chronic disease management in patients with diabetes mellitus, hypertension and hypercholesterolemia: A systematic review. *International Journal of Nursing Studies* 86, 139–150. https://doi.org/10.1016/j.ijnurstu.2018.06.016.

**Hellgren M, Wennberg P, Hedin K, Jansson S, Nilsson S, Nilsson G, Wändell P and Bengtsson Boström K** (2023) Hypertension management in primary health care: a survey in eight regions of Sweden. *Scandinavian Journal of Primary Health Care* 41(3), 343–350. https://doi.org/10.1080/02813432.2023.2242711.

**Hussain T, Allen A, Halbert J, Anderson CAM, Boonyasai RT and Cooper LA** (2015) Provider Perspectives on Essential Functions for Care Management in the Collaborative Treatment of Hypertension: The P.A.R.T.N.E.R. Framework. *Journal of General Internal Medicine* 30(4), 454–461. https://doi.org/10.1007/s11606-014-3130-4.

**John JR, Jani H, Peters K, Agho K and Tannous WK** (2020, September 2) The effectiveness of patient-centred medical home-based models of care versus standard primary care in chronic disease management: A systematic review and meta-analysis of randomised and non-randomised controlled trials. *International Journal of Environmental Research and Public Health*. MDPI AG, 1–50. https://doi.org/10.3390/ijerph17186886.

**Lee P, Pham L, Oakley S, Eng K, Freydin E, Rose T, Ruiz A, Reen J, Suleyman D, Altman V, Keating Bench K, Lee A and Mahaniah K** (2019) Using lean thinking to improve hypertension in a community health centre: A quality improvement report. *BMJ Open Quality* 8(1). https://doi.org/10.1136/bmjoq-2018-000373.

**Lin C, Peschard V-G, Kannuthurai V, Walker T, Moorehead C and Fondahn ED** (2020) Hypertension management in an internal medicine resident clinic: A quality improvement project update. In *Abstracts from the 2020 Annual Meeting of the Society of General Internal Medicine*, Vol. 35. NLM (Medline), S153–S154. https://doi.org/10.1007/s11606-020-05890-3.

**Lukewich J, Edge DS, Vandenkerkhof E, Williamson T and Tranmer J** (2018, November 1) Team composition and chronic disease management within primary healthcare practices in eastern Ontario: An application of the Measuring Organizational Attributes of Primary Health Care Survey. *Primary Health Care Research and Development*. Cambridge University Press, 622–628. https://doi.org/10.1017/S1463423618000257.

**Maheux B, Ma M, Côté L, Sobanjo O, Authier L, Fcmf MD, Lajeunesse J, Fcmf M, Leclerc M and Lefort L** (2014) Collaboration between family physicians and nurse clinicians. Opinions of graduates in family medicine. *Canadian Family Physician • Le Médecin de Famille Canadien* 60, e416–e422.

**Marco-Moreno I, Martínez-Ibañez P, Avelino-Hidalgo E, Bellot-Pujalte L, Barreira-Franch I, Martínez-Ibañez L, Escrig-Veses M, Giménez-Loreiro M, Bóveda-García M, Calleja-del-Ser M, Hurtado I, García-Sempere A, Rodríguez-Bernal CL, Peiró S, Sanfélix-Genovés J and Sanfelix-Gimeno G** (2020) Primary care doctors’ views on self-monitoring of blood pressure and self-titration among patients with uncontrolled hypertension in Spain. The ADAMPA trial focus group study. *BJGP Open* 4(4), 1–12. https://doi.org/10.3399/bjgpopen20X101062.

**Martínez-González NA, Tandjung R, Djalali S, Huber-Geismann F, Markun S and Rosemann T** (2014) Effects of physician-nurse substitution on clinical parameters: A systematic review and meta-analysis. *PLoS ONE* 9(2). https://doi.org/10.1371/journal.pone.0089181.

**Martínez-Mardones F, Benrimoj SI, Ahumada-Canale A, Plaza-Plaza JC and Garcia-Cardenas V** (2023) BC Clinical impact of medication reviews with follow-up in cardiovascular older patients in primary care: A cluster-randomized controlled trial. *British Journal of Clinical Pharmacology* 89(7), 2131–2143. https://doi.org/10.1111/bcp.15682.

**Matthys E, Remmen R and Van Bogaert P** (2017) An overview of systematic reviews on the collaboration between physicians and nurses and the impact on patient outcomes: What can we learn in primary care? *BMC Family Practice* 18(1). https://doi.org/10.1186/s12875-017-0698-x.

**Nunes Oliveira C, Galvão Oliveira M, Wildes Amorim W, Nicolaevna Kochergin C, Mistro S, de Medeiros DS, Oliveira Silva K, Moraes Bezerra V, Honorato dos Santos de Carvalho VC, Bispo Júnior JP, Louzado JA, Lopes Cortes M and Arruda Soares D** (2022) Physicians’ and nurses’ perspective on chronic disease care practices in Primary Health Care in Brazil: a qualitative study. *BMC Health Services Research* 22(1). https://doi.org/10.1186/s12913-022-08078-z.

**Ödesjö H, Adamsson Eryd S, Franzén S, Hjerpe P, Manhem K, Rosengren A, Thorn J and Björck S** (2019) Visit patterns at primary care centres and individual blood pressure level–a cross-sectional study. *Scandinavian Journal of Primary Health Care* 37(1), 53–59. https://doi.org/10.1080/02813432.2019.1569369.

**O’Neill JL, Cunningham TL, Wiitala WL and Bartley EP** (2014) Collaborative hypertension case management by registered nurses and clinical pharmacy specialists within the Patient Aligned Care Teams (PACT) model. *Journal of General Internal Medicine* 29(SUPPL. 2). https://doi.org/10.1007/s11606-014-2774-4.

**Perris K** (2020) Optimizing the RN Role in Ambulatory Care with Standardized Procedures. *Continuing Nursing Education* 3–7. www.aaacn.org

**Price-Haywood EG, Amering S, Luo Q and Lefante JJ** (2017) Clinical Pharmacist Team-Based Care in a Safety Net Medical Home: Facilitators and Barriers to Chronic Care Management. *Population Health Management* 20(2), 123–131. https://doi.org/10.1089/pop.2015.0177.

**Proia KK, Thota AB, Njie GJ, Finnie RKC, Hopkins DP, Mukhtar Q, Pronk NP, Zeigler D, Kottke TE, Rask KJ, Lackland DT, Brooks JF, Braun LT and Cooksey T** (2014) Team-based care and improved blood pressure control: A community guide systematic review. *American Journal of Preventive Medicine* 47(1), 86–99. https://doi.org/10.1016/j.amepre.2014.03.004.

**Rumball-Smith J, Wodchis WP, Koné A, Kenealy T, Barnsley J and Ashton T** (2014) Under the same roof: Co-location of practitioners within primary care is associated with specialized chronic care management. *BMC Family Practice* 15(1). https://doi.org/10.1186/1471-2296-15-149.

**Schor A, Bergovoy-Yellin L, Landsberger D, Kolobov T and Baron-Epel O** (2019) Multidisciplinary work promotes preventive medicine and health education in primary care: A cross-sectional survey. *Israel Journal of Health Policy Research* 8(1). https://doi.org/10.1186/s13584-019-0318-4.

**Stephen C, Halcomb E, Batterham M, McInnes S and Zwar** N (2023a) Impact of a general practice nurse intervention to improve blood pressure control. The ImPress Study. *Australian Journal of General Practice* 52(12).

**Stephen C, Halcomb E, Batterham M and Zwar N** (2023b) ‘Jumping on the blood pressure bandwagon’: GP, nurse and patient perspectives of a general practice nurse-leg hypertension management intervention. In *Abstracts of the Australasian Association for Academic Primary Care (AAAPC) Annual Research Conference, 17–18 August 2023*, Vol. 29. CSIRO Publishing, lxxiv–lxxv. https://doi.org/10.1071/pyv29n4abs.

**Stojnić N, Klemenc-Ketiš Z, Mori Lukančič M, Zavrnik Č and Poplas Susič A** (2023) Perceptions of the primary health care team about the implementation of integrated care of patients with type 2 diabetes and hypertension in Slovenia: qualitative study. *BMC Health Services Research* 23(1). https://doi.org/10.1186/s12913-023-09353-3.

**Van Grondelle SE, Van Bruggen S, Meijer J, Van Duin E, Bots ML, Rutten G, Vos HMM, Numans ME and Vos RC** (2022) Opinions on hypertension care and therapy adherence at the healthcare provider and healthcare system level: a qualitative study in the Hague, Netherlands. *BMJ Open* 12(7). https://doi.org/10.1136/bmjopen-2022-062128.

**Weinstein E and Rucker LM** (2016) Team-based care to improve control of hypertension in an inner city practice. *Healthcare* 4(1), 52–56. https://doi.org/10.1016/j.hjdsi.2015.10.006.

**Weise S, Steybe T, Thiel C and Frese T** (2023) Are Nurse-Led Patient Consultations and Nurse-Led Dose Adjustments of Permanent Medication Acceptable for Patients with Diabetes Mellitus and Hypertension in General Practice? – Results of a Focus Group Study. *Patient Preference and Adherence* 17, 1501–1512. https://doi.org/10.2147/PPA.S411902.

**Yu EYT, Wan EYF, Mak IL, Chao DVK, Ko WWK, Leung M, Li YC, Liang J, Luk W, Wong MMY, Ha TKH, Chan AKC, Fong DYT and Lam CLK** (2023) Assessment of Hypertension Complications and Health Service Use 5 Years After Implementation of a Multicomponent Intervention. *JAMA Network Open* 6(5), E2315064. https://doi.org/10.1001/jamanetworkopen.2023.15064.
