# Supplementary material for: Practice nurse management of hypertension in primary care: a scoping review
Source: Prim Health Care Res Dev. 2026 Aug 11;27:e89. doi: 10.1017/S1463423626101455 (PMC13740167; doi:10.1017/S1463423626101455)
Supplement: Supplementary file 4 [file S1463423626101455sup004.docx]

**Appendix 4**

**Qualitative Outcomes**

|  | (Maheux *et al.* 2014) | (Hussain *et al.* 2015) | (Marco-Moreno *et al.* 2020) | (Van Grondelle *et al.* 2022) | (Nunes Oliveira *et al.* 2022) | (Weise *et al.* 2023) | (Stephen *et al.* 2023b) | (Stojnić *et al.* 2023) |
| --- | --- | --- | --- | --- | --- | --- | --- | --- |
| Summary of study | A review of graduating GPs’ opinions on interprofessional collaboration between them and NP’s. | Review of care management functions for HTN in primary care, to help inform further study projects. | Review of GP opinion on a trial (ADAMPA) for self-monitoring of BP and self-titration of medication by patients. This is a Spanish study based on two positive UK studies (TASMINH2 and TASMINH-SR – studies prior to search history dates). | Medication non-adherence is common for those with HTN (80% do not take the medication as prescribed, or at all). This review looks at the relevance of the healthcare provider and the healthcare system on this non-adherence. | Primary health care systems provide better healthcare, particularly for chronic disease (which includes HTN). This is a review of GP’s and RN’s perspectives of the care they give. | A review of patient opinion on PN led patient consultations and medication dose adjustment for diabetes and/or HTN | A qualitative review of the staff and patients involved in the ‘Impress study’ (Stephen *et al.* 2023a) | A review of the understanding and opinions on the implementation of integrated care for T2DM and HTN, from multiple members of the primary care team |
| Type of study | Anonymous Online survey of 186 graduates. | Interviews of 47 primary care practitioners (32 GPs, six NP and one physician assistant) in six focus groups, using grounded theory and iterative coding. | Focus group methodology involving 12 GPs. | Focus group review using the theoretical domains framework and the behaviour change wheel. Involving 9 GPs, 6 PN’s and 5 secondary care physicians specialising in HTN | Semi-structured interviews of 5 GPs and 18 RN’s, looking at three dimensions: health care organisation, technical aspects of care and biopsychosocial approaches to care. | Online focus groups using semi-structured interviews, involving 17 patients who have diabetes or HTN, and were on at least one medication. Thematic analysis was used to identify topics. | Semi-structured interviews of 3 GPs, 5 RN's and 6 patients. | Focus group discussion. Eight groups, involving 48 professionals, including GP, RN, PN and community nurses. Analysis using the NVivo program. |
| Themes developed | Most GPs reported they were open to NP’s titrating medication for HTN, and some other routine medical conditions. There were many other medical conditions for which they felt it was not appropriate for them to act independently, but they could act within an agreed plan from the GP. | Themes are noted in Box 1.  Not enough time is the main issue, and patients “ cannot do it on their own”. Care management is seen as a possible solution, making sure people “don’t stop taking care of themselves” | They do not always trust the patient’s numbers, with concerns about machine calibration. Patient participation reduces anxiety – “it gives them security”. Not all patients are suitable; some appear over keen, others under, to change the dose. | More team-based care is required, both in the primary care team (including pharmacists and social workers) and the primary and secondary care teams. This should involve better collaboration as well as education and support. IT systems are seen as helpful in this. Staff struggle with ‘optimism’ when patients do not progress. | Issues of communication between primary and secondary care were noted. Collaborative care is felt best achieved through horizontal work organisation and shared decision-making. | The improved access was seen as positive – “you can get to the nurse faster than to the doctor”. The decision was personal – “I trust them [PN] even more than the doctor in some points”. PN were seen as positive agents for change. It was seen to help free up GP time for more complex issues. | Discussions were around three themes – “the need for change”, “ the challenge for change” and “sustaining change”. | 12 main themes around accessibility, cooperation and preventative healthcare. Concerns were about the high workload, lack of workforce and patient ambivalence about the seriousness of the conditions. It was felt that patients should be given more responsibility, as patients appear to be feeling the system should be taking care of them. HTN care requires engagement from patients, the community, the care team and the state. |
| Independent Nursing Practice Supported? | Yes, but only for NP’s, for which independent practice is part of their expected work | Not discussed | Some GPs felt nurses were vital, others pointless, in supporting patients with their hypertension. | Not discussed. | Not discussed. | Yes. Patients were generally happy with PN's independent management of multiple conditions, including medication changes, for conditions such as stable chronic conditions, including antihypertensive drugs (Fig. 1).. | By the study itself, but no data on patient or staff opinions in this report. | Current level of PN remit not discussed, and significant GP concern about advanced roles expressed. |
| Nurse and Clinical Pharmacist Team supported? | Not discussed | Not discussed | Not discussed | Not discussed. The increased use of pharmacists is supported; there are some negative issues around changing medication brands due to policy and costs (Table 4 – Q14), but their support in engagement and compliance is noted in the conclusion, where they can check for script collection. | Not discussed. | Not discussed | Not discussed. | Not discussed formally, but it is noted that even pharmacy technicians can be helpful in supporting patients with advice. There are some concerns that the pharmaceutical industry is getting more market-oriented and not patient-focused. |
| Nurse and ANP Team supported? | Not discussed. This is about GP opinion of NP independent practice, not NP oversight of PN activity. | Not discussed | Not discussed | Not discussed. The use of an ANP in titrating medication is suggested in the conclusion. | Not discussed. | Not discussed. NP’s do not appear to be part of the German health care team. | Not discussed. | Not discussed. NP’s do not appear to be part of the Slovenian health care team. |
| Nurse and GP Team supported? | Not discussed. | Not specifically discussed, but the focus of the work is looking at how a care manager (likely a nurse) can support the patient. | Some see Nurses as essential, others irrelevant – “I don’t count on nursing staff at all”. There is no discussion on nurses running the programme if it was rolled out. | Division of tasks between the GP and PN was noted to be difficult (Q4). Time with patients is seen as too short to elicit lifestyle changes (Table 4 – Q13) | Not specifically discussed. Some discussion about roles but no defining of the activities/comparisons  /plans. | Yes. For some people the clinical oversight/supervision / guidelines are required for PN first contact (Fig 2). | Not discussed. | Not discussed in relation to the question. |
| Is team-based care superior or inferior to GP care? | Not discussed. | It is felt the evidence supports this. | Not part of the question, however, the patient, as part of the team, was not part of my proposal, which perhaps it should have been. The discussion reports that poor organisational design in primary care is causing significant heterogeneity in roles and causing conflict between doctors and RN’s. | Not part of the question, but there seems to be agreement that a team-based approach is required. Although it is not defined, broader referral options are wanted – health coaches for lifestyle changes, social services for those in debt who cannot afford the medication. | Not discussed. | Positives and negatives were noted. Positives were improved access, increased time for consultations, and improved compliance due to the improved support. Concerns were increased resistance to see the GP, GP jobs being cut, PN requiring increased pay or struggling with the extra workload. | Not discussed. | It is noted that studies suggest this, and this is therefore seen as something to aspire to. This team-based care is presented (although very little detail is given) as hierarchical, with GP as care coordinator. The concept of *expanding* roles is only mentioned in connection to GP concerns about this. Nursing roles seem defined and fixed. Discussion is more about adding more roles to support the team. |
| Are there concerns about clinical safety? | Yes – by GPs for NP’s managing mental health medication independently, among others. | There are concerns about the current risk due to poor management. | None were explicitly expressed. It is noted that prior to this trial, patients were not educated on their condition, medication, or encouraged to self-manage in any formal way – such as reducing the dose when unwell or in very hot weather. | There is concern about the current state, as patients are not being optimally treated, so they are at increased CV risk. Also, those who are non-compliant are being referred to secondary care for intensive treatment when routine treatment has not actually been taken. | Not discussed. | Some patients were concerned about treatment errors – “I don’t see how I can let the nurse mess around with my medication”. There were concerns that this would lead to barriers to speaking to the GP, a lack of clarity about the roles, or that there would be fewer GPs employed. | Not discussed | Not discussed. |
| Is there support for change from clinical staff? | No – GP support is limited to specific clinical presentations. | Yes | There is, but the conclusion notes that this may be due to the positive outcomes from the study, in that self-managed BP was better managed, and therefore this opinion may not be present in other GPs. | There is no discussion that GPs should allow other members of the team to diagnose and treat HTN. | Not relevant. This is not looking at clinical care, but the environment of care for all types of chronic disease. | Not discussed. | Not discussed | Yes – but the pace is slow – “The system is such that 10 years pass before anything moves”. While expansion of nursing roles is not discussed, the use of lay people as support for chronic conditions is noted. |
| Are patient voices present? | No | Not specifically, but there is awareness of the complexity of people’s lives and the factors that could affect treatment compliance outside of the patient’s control. | No. There is a plan for further study looking at Nurses' and patients’ perspectives of the trial. | Patients and the public were not involved, as this study was looking at the healthcare aspect of medication non-adherence | No | Fully. But small numbers. | By including patients, yes, but no information on opinions. | No |
| Is there support for change from patients? | Not discussed. | Not discussed. | Not discussed. | Not discussed.  It is noted that encouraging patients to be compliant with a symptom-free disease is difficult | Not discussed. | Yes – but some caveats. | Not discussed. | Not discussed. |
| Relevance to the question. | None. The scope of practice that GPs approve of for NP is already part of their job role. This is not looking at them as supportive of the wider nursing team or PN independent activity. | Only in the agreement that time and more support for patients with HTN is needed, and this ‘care management’ role is likely to be done by a nurse, but could equally be done by an HCA. | It could be argued that Nurses or Pharmacists would be well placed to support patients with their own medication titration, much as Nurses do for diabetes and asthma at present.. Any suggestion that there is increased risk when patients are more effective than GP’s at achieving BP targets would appear to be unsupported. | A study is reported showing that only 20% of patients are fully compliant with their medication, and 60% are already self-titrating, which supports Marco-Moreno et al. | None | Very relevant. One of the few patient-focused investigations, and clearly defining the role as a PN and not the ANP. Generally supportive, with a clear remit in Fig 1 that would exceed that commonly found in the UK, with antibiotic / analgesic provision independently. | No information provided – assumed preliminary report to the AAAPC conference before final publication produced. | Nursing roles are defined, but there is no exploration of remit/independence. The fact that most care is protocolled is noted. Good collaboration between the team, with good communication tools, were seen as vital to team functioning. Nurses were seen as a link between the patient and the GP, but there is no exploration of them taking over aspects of patient care. GP’s were very concerned about the ANP role as seen in the UK, and see it as a threat to their profession. However, the very clear roles present result in high levels of trust between the GP’s and the entire nursing team. |

The abbreviation ‘GP’ is used to denote someone with a medical degree who has qualified in family medicine / primary care.

The abbreviation ‘PN’ is used to denote a Practice Nurse, or any person registered as a Nurse in that country, working in primary care, that does not have additional qualifications to allow for independent practice or hold prescribing rights, but can have some postgraduate training in their role.

The abbreviation ‘NP’ is used to denote any registered nurse who has additional qualifications to allow for independent practice and holds independent prescribing rights.

The abbreviation ‘CP’ is used to denote a Clinical Pharmacist, or a person registered as a pharmacist in that country that has additional independent prescribing rights.

The abbreviation ‘RN’ is used to denote a Registered Nurse.

The abbreviation ‘CVD’ is used to refer to cardiovascular disease.

The abbreviation ‘HTN’ is used to refer to hypertension

**References**

**Hussain T, Allen A, Halbert J, Anderson CAM, Boonyasai RT and Cooper LA** (2015) Provider Perspectives on Essential Functions for Care Management in the Collaborative Treatment of Hypertension: The P.A.R.T.N.E.R. Framework. *Journal of General Internal Medicine* 30(4), 454–461. https://doi.org/10.1007/s11606-014-3130-4.

**Maheux B, Ma M, Côté L, Sobanjo O, Authier L, Fcmf MD, Lajeunesse J, Fcmf M, Leclerc M and Lefort L** (2014) Collaboration between family physicians and nurse clinicians. Opinions of graduates in family medicine. *Canadian Family Physician • Le Médecin de Famille Canadien* 60, e416–e422.

**Marco-Moreno I, Martínez-Ibañez P, Avelino-Hidalgo E, Bellot-Pujalte L, Barreira-Franch I, Martínez-Ibañez L, Escrig-Veses M, Giménez-Loreiro M, Bóveda-García M, Calleja-del-Ser M, Hurtado I, García-Sempere A, Rodríguez-Bernal CL, Peiró S, Sanfélix-Genovés J and Sanfelix-Gimeno G** (2020) Primary care doctors’ views on self-monitoring of blood pressure and self-titration among patients with uncontrolled hypertension in Spain. The ADAMPA trial focus group study. *BJGP Open* 4(4), 1–12. https://doi.org/10.3399/bjgpopen20X101062.

**Nunes Oliveira C, Galvão Oliveira M, Wildes Amorim W, Nicolaevna Kochergin C, Mistro S, de Medeiros DS, Oliveira Silva K, Moraes Bezerra V, Honorato dos Santos de Carvalho VC, Bispo Júnior JP, Louzado JA, Lopes Cortes M and Arruda Soares D** (2022) Physicians’ and nurses’ perspective on chronic disease care practices in Primary Health Care in Brazil: a qualitative study. *BMC Health Services Research* 22(1). https://doi.org/10.1186/s12913-022-08078-z.

**Stephen C, Halcomb E, Batterham M, McInnes S and Zwar N** (2023a) Impact of a general practice nurse intervention to improve blood pressure control. The ImPress Study. *Australian Journal of General Practice* 52(12).

**Stephen C, Halcomb E, Batterham M and Zwar N** (2023b) ‘Jumping on the blood pressure bandwagon’: GP, nurse and patient perspectives of a general practice nurse-leg hypertension management intervention. In *Abstracts of the Australasian Association for Academic Primary Care (AAAPC) Annual Research Conference, 17–18 August 2023*, Vol. 29. CSIRO Publishing, lxxiv–lxxv. https://doi.org/10.1071/pyv29n4abs.

**Stojnić N, Klemenc-Ketiš Z, Mori Lukančič M, Zavrnik Č and Poplas Susič A** (2023) Perceptions of the primary health care team about the implementation of integrated care of patients with type 2 diabetes and hypertension in Slovenia: qualitative study. *BMC Health Services Research* 23(1). https://doi.org/10.1186/s12913-023-09353-3.

**Van Grondelle SE, Van Bruggen S, Meijer J, Van Duin E, Bots ML, Rutten G, Vos HMM, Numans ME and Vos** RC (2022) Opinions on hypertension care and therapy adherence at the healthcare provider and healthcare system level: a qualitative study in the Hague, Netherlands. *BMJ Open* 12(7). https://doi.org/10.1136/bmjopen-2022-062128.

**Weise S, Steybe T, Thiel C and Frese T** (2023) Are Nurse-Led Patient Consultations and Nurse-Led Dose Adjustments of Permanent Medication Acceptable for Patients with Diabetes Mellitus and Hypertension in General Practice? – Results of a Focus Group Study. *Patient Preference and Adherence* 17, 1501–1512. https://doi.org/10.2147/PPA.S411902.
